# Exploring active ingredients and function mechanisms of Ephedra-bitter almond for prevention and treatment of Corona virus disease 2019 (COVID-19) based on network pharmacology

**DOI:** 10.1186/s13040-020-00229-4

**Published:** 2020-11-10

**Authors:** Kai Gao, Yan-Ping Song, Anna Song

**Affiliations:** 1 Pharmacy College, Shaanxi University of Chinese Medicine, Xianyang, Shaanxi China; 2 Shaanxi Academy of Traditional Chinese Medicine, Xi’an, Shaanxi China; 3 Michigan State University, East Lansing, MI USA

**Keywords:** COVID-19, Ephedra-bitter almond, Molecular docking simulation, Traditional Chinese medicine, Molecular mechanisms of pharmacological action

## Abstract

**Background:**

COVID-19 has caused a global pandemic, and there is no wonder drug for epidemic control at present. However, many clinical practices have shown that traditional Chinese medicine has played an important role in treating the outbreak. Among them, ephedra-bitter almond is a common couplet medicine in anti-COVID-19 prescriptions. This study aims to conduct an exploration of key components and mechanisms of ephedra-bitter almond anti-COVID-19 based on network pharmacology.

**Material and methods:**

We collected and screened potential active components of ephedra-bitter almond based on the TCMSP Database, and we predicted targets of the components. Meanwhile, we collected relevant targets of COVID-19 through the GeneCards and CTD databases. Then, the potential targets of ephedra-bitter almond against COVID-19 were screened out. The key components, targets, biological processes, and pathways of ephedra-bitter almond anti-COVID-19 were predicted by constructing the relationship network of herb-component-target (H-C-T), protein-protein interaction (PPI), and functional enrichment. Finally, the key components and targets were docked by AutoDock Vina to explore their binding mode.

**Results:**

Ephedra-bitter almond played an overall regulatory role in anti-COVID-19 via the patterns of multi-component-target-pathway. In addition, some key components of ephedra-bitter almond, such as β-sitosterol, estrone, and stigmasterol, had high binding activity to 3CL and ACE2 by molecular docking simulation, which provided new molecular structures for new drug development of COVID-19.

**Conclusion:**

Ephedra-bitter almonds were used to prevent and treat COVID-19 through directly inhibiting the virus, regulating immune responses, and promoting body repair. However, this work is a prospective study based on data mining, and the findings need to be interpreted with caution.

**Supplementary Information:**

The online version contains supplementary material available at 10.1186/s13040-020-00229-4.

## Background

The recent emergence of the severe acute respiratory syndrome coronavirus 2 (SARS-CoV-2) has resulted in a WHO-declared public health emergency of international concern [1]. Since December 2019, the sudden epidemic of Corona Virus Disease 2019 (COVID-19) has seriously threatened the healthy life of the people. As of April 3, 2020, there have been approximately 1,020,000 confirmed cases of COVID-19 worldwide, with 54,000 deaths. Among them, a total of 82,813 cases of COVID-19 (including overseas imported cases) were diagnosed in China, with 76,791 cured cases and 3331 deaths. From this data, it is not difficult to find that China’s prevention and control of COVID-19 epidemic are beginning to bear fruit, which is not only due to measures such as restricting the flow of people and vigorously publicizing but also to the important factor of medical assistance. In particular, the widespread use of Traditional Chinese Medicine (TCM) has played a huge role in the prevention and control of this epidemic [2].

During the epidemic, the administrations of TCM and health committees of various provinces and cities in China have formulated and issued Chinese medicine prevention and treatment programs for COVID-19 based on over-all symptoms of the patients. Some studies [3, 4] have explored the law of drug use through data mining on these prescriptions for the prevention and treatment of COVID-19, and found that ephedra (Ephedrae Herba) - bitter almond (Armeniacae Semen Amarum) is one of the most frequently used couplet medicines (called “yaodui” in Chinese, this is a relatively fixed combination of two herbs commonly used in TCM). For example, Qingfei Paidu Decoction, as a recommended prescription in “the Diagnosis and Treatment Protocol for Novel Coronavirus Pneumonia” formulated by the National Health Commission of the People’s Republic of China [5], contains the couplet medicines of ephedra-bitter almond, and the effective cure rate of this prescription for COVID-19 is more than 90% [6]. When it was used as an adjuvant treatment to western medicine, it could relieve symptoms, improve the regression of lung inflammation, and show a tendency to reduce the degree of multi-organ impairment [7]. However, due to the complexity of TCM components and the limitations of the current experimental conditions, we cannot know the pharmacological substance basis and mechanism of ephedra-bitter almond against COVID-19.

Traditionally, the hypothesis that “one drug for one target for one disease” has influenced various aspects of drug discovery strategies. However, advances in systems biology have shown that interventions with a single node may not be effective in the treatment of complex diseases [8]. Hence, network biology seems to clarify our understanding of drug action more clearly. Network pharmacology is a cross-discipline which is based on systems biology and combines polypharmacology, molecular network data, bioinformatics, and computer simulation [9–11]. This discipline has resulted in widespread public interest, and humanity has made extraordinary progress in further understanding diseases through this knowledge and technology over recent years. Some studies try to redefine diseases based on the methods and ideas of network pharmacology, and provide new ideas for the treatment of diseases and the reuse of drugs [12]. For example, Feixiong Cheng et al. identified new drug-disease associations for old drugs by quantifying the proximity of disease genes and drug targets in the protein-protein interaction (PPI) network [13]; they also proposed a network-based method to identify clinically effective drug combinations for specific diseases, with a view to discovering the synergistic and attenuating rules of drug compatibility [14]. Excitingly, during the COVID-19 pandemic people used methods such as system pharmacology and network medicine to quickly identify reusable drugs and potential drug combinations for SARS-CoV-2 from candidate drugs [15, 16]. Compared with the traditional model of developing new drugs from scratch, this method can minimize the conversion gap between preclinical test results and clinical results, and facilitate the rapid formulation of effective treatment strategies for the epidemic.

**Fig. 1:**
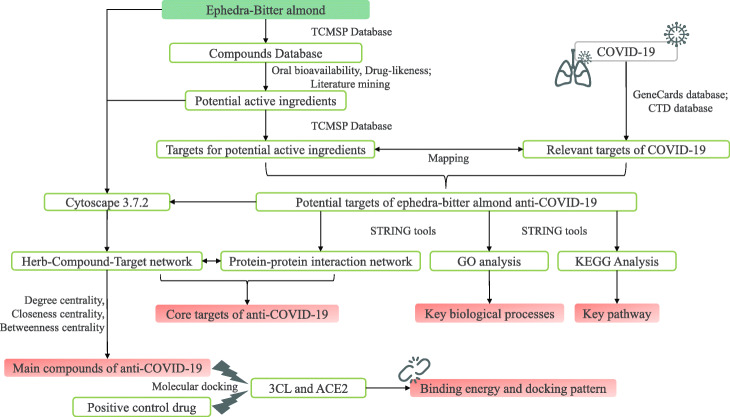
Research process

In recent years, network pharmacology has become more and more widely used in the research of TCM formulations, including targets discovery, bioactive compounds screening, toxicity evaluation, mechanism research, and quality control research, and has also derived new frontier disciplines such as network toxicology and TCM-Gut microbiota network pharmacology [17]. It is considered to be one of the most suitable techniques for TCM formulation research [18]. By constructing a complex network of herbs-components-targets-disease, the action mechanism of TCM prescriptions can be explained from a system level, which is consistent with the “holistic view” of TCM for treating diseases. Its ability to analyze and process huge data information provides a powerful tool for the analysis of the overall action mechanism of TCM prescription. In this study, we explored the potential pharmacodynamic material basis and molecular mechanism of ephedra-bitter almond against COVID-19 using network pharmacology and molecular docking technology, to provide some basis for its experimental research (Fig. 1).

## Material and methods

### Screening of potentially active components from ephedra-bitter almond

All candidate compounds of ephedra and bitter almond were collected from the TCMSP Database [19] (Traditional Chinese Medicine Systems Pharmacology Database and Analysis Platform, http://tcmspw.com/tcmsp.php), a unique systems pharmacology platform of Chinese herbal medicines that captures the relationships between drugs, targets, and diseases.

Oral bioavailability (OB) is a significant indicator of the efficiency of drug or active compound delivery to the body’s blood circulation [20]. Drug-likeness (DL) means “molecule which holds functional groups and/or has physical properties consistent with the majority of known drugs” [21]. Therefore, for new drug candidates, good OB and DL are one of their most important pharmacokinetic parameters. In the TCMSP database, the OB values of each compound were predicted by the OBioavail 1.1 model, which was built based on 805 drug-like molecules of known human OB [22]. The DL values of each compound were predicted based on a construction model of molecular descriptors and Tanimoto coefficients. These models have been shown to be effective in screening drugs or active ingredients [23, 24]. In this study, we selected compounds with OB ≥ 30% and DL ≥ 0.18 as candidate ingredients.

In addition, in order to avoid filtering out the effective ingredients with exact curative effects of ephedra and bitter almond in the screening process, we also supplemented the active ingredients by means of literature research.

### Prediction and screening of targets

Target prediction of chemical composition is essential to elucidate its pharmacological effects. Therefore, this study predicted the potential targets of the components through the TCMSP database [19]. The Weighted Ensemble Similarity method and support vector machine method were applied to the target prediction in the TCMSP database. The model showed better prediction performance of drug-target interaction, with a consistency of 82.83%, a sensitivity of 81.33%, and a specificity of 93.62% [25, 26], which had been widely proven to be effective [27]. The predicted target proteins were imported into the UniProt database (The Universal Protein Resource, http://www.uniprot.org/), and the species was set to “*Homo sapiens*” to standardize its name. Meanwhile, we used “coronavirus disease 2019” OR “COVID-19” as keywords to search the GeneCards database [28] (https://www.genecards.org/) and the CTD database (Comparative Toxicogenomics Database, http://ctdbase.org/) to get the relevant targets of COVID-19. Then, based on the mapping analysis of the components targets and the disease targets, the potential action targets of ephedra-bitter almond against COVID-19 were screened out.

### Construction of “herb-component-target” network

In order to investigate the multi-component and multi-target therapeutic feature of ephedra-bitter almond in combating COVID-19, we completed a relationship mapping of the herbs of ephedra-bitter almond, the candidate compounds, and potential targets using Cytoscape 3.7.2, a standard tool for biological network visualization and data integration [29, 30]. The herb-component-target (H-C-T) network was constructed in this work. In addition, we analyzed the quantitative properties of the network using plug-ins in the Cytoscape Network Analyzer [31]. Through the parameters such as degree centrality (DC), closeness centrality (CC), and betweenness centrality (BC), we screened out the potential pharmacodynamic material basis and main action targets of ephedra-bitter almond against COVID-19. Centrality is an important parameter to describe the characteristics of network topology. DC is the sum of the number of nodes directly connected to a node, which is the most direct indicator of the importance of nodes in network analysis. CC is the sum of distances from one node to all other nodes, reflecting the extent of the proximity of a node to other nodes. BC is the number of shortest paths through a node, which reflects the degree of cohesion of nodes in the network. In this study, if the three-parameter values ​​of a node were greater than the corresponding median, then the node was considered to have an important position in the network, and the substance represented by the node was the key component or target.

### Construction of protein-protein interaction (PPI) network

We imported the potential targets of ephedra-bitter almond anti-COVID-19 into the STRING tool (Search Tool for Retrieval of Interacting Genes/Proteins, https://string-db.org/) [32], and set the network edge in Basic Settings to confidence; the active interaction sources were set to Textmining, Experiments, Databases, Co-expression, Neighborhood, Gene Fusion, and Co-occurrence; minimum required interaction score was set to highest confidence 0.900 for PPI analysis. Then, the interaction information was further visually analyzed by Cytoscape, and the main targets were predicted by the parameters such as DC, CC, and BC. Combined with the main targets predicted by H-C-T network analysis in the previous step, the core targets of ephedra-bitter almond against COVID-19 were obtained.

### Gene ontology (GO) and Kyoto encyclopedia of genes and genomes (KEGG) analysis

We imported the potential targets of ephedra-bitter almond anti-COVID-19 into the STRING database for enrichment analysis of GO and KEGG pathways [32], aiming to explore the relevant biological processes and signal pathways of potential anti-COVID-19 targets. After selecting the GO and KEGG terms with False Discovery Rate (*FDR*) ≤ 0.05, the top 10 terms and top 15 pathways with the highest number of observed genes were selected, and the bubble diagrams were drawn by R statistical modeling software to further analyze and predict the key pathways of ephedra-bitter almond anti-COVID-19.

### Molecular docking prediction

Currently, it is generally believed that 3CL hydrolase (Mpro) from 2019-nCoV coronavirus and angiotensin-converting enzyme 2 (ACE2) from host cells are potential targets for anti-COVID-19 [33, 34]. Therefore, we performed molecular docking simulation between the key components based on H-C-T network analysis and the target proteins.

We searched and downloaded the protein structure of 3CL hydrolase (PDB ID: 6 LU7) and ACE2 (PDB ID: 1R42) from Protein Data Bank (PDB, http://www.rcsb.org), and the 3D structure of key components from ZINC database (http://zinc.docking.org/), respectively. The protein structure and active components were modified by Pymol and AutoDockTools [35, 36]. The processed protein and components were introduced into AutoDock Vina for molecular docking, and the binding energy was calculated using the Iterated Local Search global optimizer [37]. The cluster with the maximum absolute value of binding energy was selected from the docking results, and the lowest value in the cluster was the binding energy between the component and the protein molecule.

Meanwhile, we selected hydroxychloroquine, which is a potential antiviral drug for anti-COVID-19, as a positive control [38], and downloaded its structure. The molecular docking simulation was performed under the same conditions, and the obtained binding energy was compared with the active components of ephedra-bitter almond.

## Results

### Potential active components of ephedra-bitter almond

In the TCMSP Database, we collected a total of 363 candidate components from ephedra, and a total of 113 candidate components from bitter almond. Through observation and analysis, it was found that ephedra mainly contains alkaloids, flavonoids, volatile oils, and organic acids, while bitter almonds mainly contain glycosides, fatty oils, proteins, and amino acids.

Next, we screened potential active components based on their ADME properties. As a result, 40 potential active molecules with OB ≥ 30% and DL ≥ 0.18 were obtained, accounting for 9.37% of the total. In addition, although the OB or DL values of some compounds do not meet the screening criteria, the literature shows that they have related pharmacological activities and belong to higher content and more typical components in the herb. Therefore, in order to obtain more accurate results, these compounds were also included in the candidate compounds for further analysis. For instance, amygdalin with relatively small OB value was retained for further analysis since it was an active ingredient of bitter almond [39]. It has been reported that amygdalin may play a protective effect on acute lung injury by inhibiting NF-κB and NLRP3 signal pathways [40]. Furthermore, the sympathomimetic effect of alkaloids in ephedra was the material basis for the pharmacological effects of ephedra [41], so we also included ephedrine, pseudoephedrine, norephedrine, norpseudoephedrine, methylephedrine, and (+)-N-Methylpseudoephedrine in this study. Finally, we screened a total of 47 “potentially active compounds” (Table 1).

**Table 1:** Basic information of 47 potentially active compounds obtained by ADME **screening**

No.	Mol ID	Molecule Name	OB (%)	DL	Herb
1	MOL000006	luteolin	36.16	0.25	Ephedra
2	MOL000098	quercetin	46.43	0.28	Ephedra
3	MOL000358	β-sitosterol	36.91	0.75	Ephedra
4	MOL000422	kaempferol	41.88	0.24	Ephedra
5	MOL001494	ethyl linoleate	42	0.19	Ephedra
6	MOL001506	squalene	33.55	0.42	Ephedra
7	MOL001755	Stigmast-4-en-3-one	36.08	0.76	Ephedra
8	MOL001771	clionasterol	36.91	0.75	Ephedra
9	MOL002823	herbacetin	36.07	0.27	Ephedra
10	MOL002881	diosmetin	31.14	0.27	Ephedra
11	MOL004328	naringetol	59.29	0.21	Ephedra
12	MOL004576	taxifolin	57.84	0.27	Ephedra
13	MOL004798	delphinidin	40.63	0.28	Ephedra
14	MOL005043	campesterol	37.58	0.71	Ephedra
15	MOL005190	eriodictyol	71.79	0.24	Ephedra
16	MOL005573	genkwanin	37.13	0.24	Ephedra
17	MOL005842	pectolinarigenin	41.17	0.3	Ephedra
18	MOL006594	ephedrine	43.35	0.03	Ephedra
19	MOL006637	pseudoephedrine	52.25	0.03	Ephedra
20	MOL007214	(+)-leucocyanidin	37.61	0.27	Ephedra
21	MOL009189	methylephedrine	44.08	0.04	Ephedra
22	MOL009190	norephedrine	66.05	0.03	Ephedra
23	MOL009191	norpseudoephedrine	68.94	0.03	Ephedra
24	MOL009194	(+)-N-Methylpseudoephedrine	37.12	0.04	Ephedra
25	MOL010489	leucocianidol	30.84	0.27	Ephedra
26	MOL010788	leucopelargonidin	57.97	0.24	Ephedra
27	MOL011319	Butyl octyl phthalate	43.74	0.24	Ephedra
28	MOL000449	stigmasterol	43.83	0.76	Ephedra, Bitter almond
29	MOL000492	cianidanol	54.83	0.24	Ephedra, Bitter almond
30	MOL000211	betulinic acid	55.38	0.78	Bitter almond
31	MOL000359	sitosterol	36.91	0.75	Bitter almond
32	MOL000953	cholesterol	37.87	0.68	Bitter almond
33	MOL001320	amygdalin	4.42	0.61	Bitter almond
34	MOL002211	11,14-eicosadienoic acid	39.99	0.2	Bitter almond
35	MOL002311	glycyrol	90.78	0.67	Bitter almond
36	MOL002372	(E,E,E,E)-Squalene	33.55	0.42	Bitter almond
37	MOL003410	Ziziphin_qt	66.95	0.62	Bitter almond
38	MOL004355	α-spinasterol	42.98	0.76	Bitter almond
39	MOL004841	licochalcone B	76.76	0.19	Bitter almond
40	MOL004903	liquiritin	65.69	0.74	Bitter almond
41	MOL004908	glabridin	53.25	0.47	Bitter almond
42	MOL005017	phaseol	78.77	0.58	Bitter almond
43	MOL005030	11-Eicosenoic acid	30.7	0.2	Bitter almond
44	MOL007207	(R)-coclaurine	79.64	0.24	Bitter almond
45	MOL010921	estrone	53.56	0.32	Bitter almond
46	MOL010922	butanedioic acid	31.62	0.23	Bitter almond
47	MOL012922	l-Stepholidine	87.35	0.54	Bitter almond

### Target prediction and disease mapping

In this study, a total of 243 targets of the compounds were predicted by the TCMSP database (after deduplication). Meanwhile, a total of 5329 targets related to COVID-19 were obtained in the GeneCards database and the CTD database. Then, the compound targets were mapped to COVID-19-related targets. After screening, 178 targets related to herbal ingredients were identified. These targets were potential targets of ephedra-bitter almond against COVID-19 (Table 2).

**Table 2:** The information on potential targets of ephedra-bitter almond against COVID-19

No.	UniProt ID	Gene Symbol	No.	UniProt ID	Gene Symbol	No.	UniProt ID	Gene Symbol
1	P80404	ABAT	61	Q9NRD8	DUOX2	121	P01106	MYC
2	P33527	ABCC1	62	Q01094	E2F1	122	P14598	NCF1
3	Q9UNQ0	ABCG2	63	Q14209	E2F2	123	Q15788	NCOA1
4	Q13085	ACACA	64	P01133	EGF	124	Q15596	NCOA2
5	P22303	ACHE	65	P00533	EGFR	125	Q16236	NFE2L2
6	Q08462	ADCY2	66	P19419	ELK1	126	P25963	NFKBIA
7	Q15848	ADIPOQ	67	P04626	ERBB2	127	P35228	NOS2
8	P08913	ADRA2A	68	P21860	ERBB3	128	P29474	NOS3
9	P07550	ADRB2	69	P03372	ESR1	129	P15559	NQO1
10	P35869	AHR	70	Q92731	ESR2	130	O75469	NR1I2
11	O95433	AHSA1	71	P13726	F3	131	P08235	NR3C2
12	P31749	AKT1	72	P49327	FASN	132	Q9BZD4	NUF2
13	P09917	ALOX5	73	P01100	FOS	133	P11926	ODC1
14	P04114	APOB	74	P17302	GJA1	134	P09874	PARP1
15	P10275	AR	75	P49841	GSK3B	135	P12004	PCNA
16	Q92934	BAD	76	P00390	GSR	136	P06401	PGR
17	Q07812	BAX	77	P09488	GSTM1	137	P11309	PIM1
18	P10415	BCL2	78	P09211	GSTP1	138	P00750	PLAT
19	Q07817	BCL2L1	79	Q16665	HIF1A	139	P00749	PLAU
20	Q9BXY8	BEX2	80	P52789	HK2	140	P27169	PON1
21	O15392	BIRC5	81	P04035	HMGCR	141	P16435	POR
22	P00918	CA2	82	P09601	HMOX1	142	Q07869	PPARA
23	P42574	CASP3	83	Q00613	HSF1	143	Q03181	PPARD
24	P55210	CASP7	84	P07900	HSP90AA1	144	P37231	PPARG
25	Q14790	CASP8	85	P11021	HSPA5	145	P17252	PRKCA
26	P55211	CASP9	86	P04792	HSPB1	146	P05771	PRKCB
27	P04040	CAT	87	P05362	ICAM1	147	O43242	PSMD3
28	Q03135	CAV1	88	P01579	IFNG	148	P43115	PTGER3
29	P13500	CCL2	89	P01344	IGF2	149	P35354	PTGS2
30	P20248	CCNA2	90	P17936	IGFBP3	150	P06400	RB1
31	P14635	CCNB1	91	O14920	IKBKB	151	Q04206	RELA
32	P24385	CCND1	92	P22301	IL10	152	Q06455	RUNX1T1
33	P29965	CD40LG	93	P01583	IL1A	153	Q13950	RUNX2
34	P06493	CDK1	94	P01584	IL1B	154	P19793	RXRA
35	P24941	CDK2	95	P60568	IL2	155	Q14524	SCN5A
36	P11802	CDK4	96	P05112	IL4	156	P16581	SELE
37	P38936	CDKN1A	97	P05231	IL6	157	P05121	SERPINE1
38	O14757	CHEK1	98	P06213	INSR	158	P14672	SLC2A4
39	O96017	CHEK2	99	P10914	IRF1	159	P23975	SLC6A2
40	Q15822	CHRNA2	100	P05412	JUN	160	Q01959	SLC6A3
41	O15111	CHUK	101	Q12809	KCNH2	161	P31645	SLC6A4
42	O14493	CLDN4	102	P35968	KDR	162	P35610	SOAT1
43	P02452	COL1A1	103	P01130	LDLR	163	P00441	SOD1
44	P02461	COL3A1	104	P21397	MAOA	164	P10451	SPP1
45	P02741	CRP	105	P27338	MAOB	165	P36956	SREBF1
46	P17538	CTRB1	106	P11137	MAP2	166	P42224	STAT1
47	P07339	CTSD	107	P28482	MAPK1	167	P01137	TGFB1
48	P02778	CXCL10	108	Q16539	MAPK14	168	P07204	THBD
49	P19875	CXCL2	109	P27361	MAPK3	169	P01375	TNF
50	P10145	CXCL8	110	P45983	MAPK8	170	P11387	TOP1
51	P11511	CYP19A1	111	Q07820	MCL1	171	P11388	TOP2A
52	P04798	CYP1A1	112	Q00987	MDM2	172	P04637	TP53
53	P05177	CYP1A2	113	P08581	MET	173	P14679	TYR
54	Q16678	CYP1B1	114	O43451	MGAM	174	Q9HAW9	UGT1A8
55	P08684	CYP3A4	115	P03956	MMP1	175	P19320	VCAM1
56	Q96PD7	DGAT2	116	P08253	MMP2	176	P15692	VEGFA
57	P27487	DPP4	117	P08254	MMP3	177	P47989	XDH
58	P21728	DRD1	118	P14780	MMP9	178	P98170	XIAP
59	P14416	DRD2	119	P05164	MPO			
60	P35462	DRD3	120	P55157	MTTP			

### Network construction

**Fig. 2:**
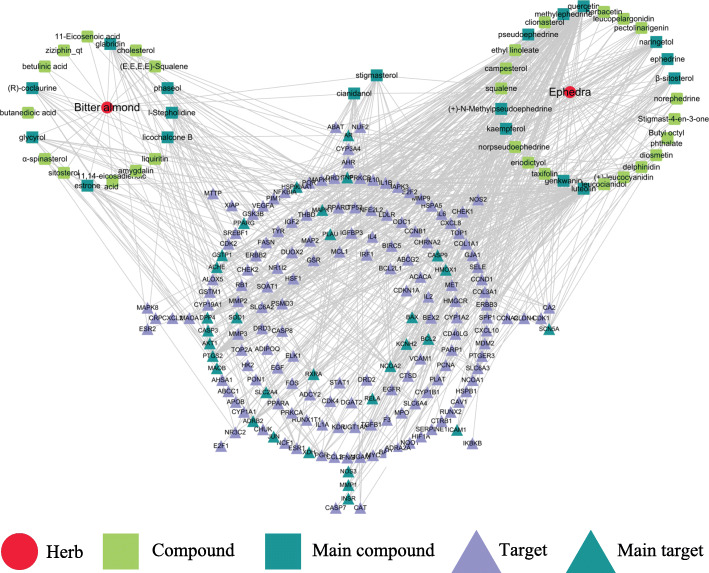
Herb-compound-target (H-C-T) network of ephedra-bitter almond against COVID-19. The red circles represent the herbs, the green squares represent the compounds and the blue squares represent main compounds, the purple triangles represent the potential targets and the blue triangles represent main potential targets

In this step, the H-C-T network was constructed including 227 nodes (2 herbs, 47 active compounds, and 178 potential targets) and 569 edges (Fig. 2). The circles represent herbs, the squares represent compounds and the triangles represent the potential targets of ephedra-bitter almond against COVID-19. The DC, CC, and BC values of compound nodes and target nodes were shown in Supplementary Material Table S1 and Table S2, respectively. The results show that the compound nodes had a median DC value of 7, a median CC of 0.3565, and a median BC of 0.0033. Among them, the three centralities of 19 compounds are higher than the median, including quercetin, luteolin, kaempferol, naringetol, β-sitosterol, glabridin, stigmasterol, licochalcone B, cianidanol, ephedrine, pseudoephedrine, phaseol, methylephedrine, (+)-N-Methylpseudoephedrine, l-Stepholidine, estrone, glycyrol, genkwanin and (R)-coclaurine, which indicates that these compounds play a relatively important role in the network. They may be the main active components of ephedra-bitter almond to exert anti-COVID-19.

Also, the target nodes have a median DC value of 2, a median CC of 0.3628, and a median BC of 0.0001. Among them, the three centralities of 31 targets are higher than the median, including PTGS2, HSP90AA1, AR, PPARG, DPP4, ACHE, NCOA2, NOS3, RELA, AKT1, BCL2, BAX, TNF, JUN, CASP3, XDH, MMP1, HMOX1, ICAM1, GSTP1, SLC2A4, INSR, KCNH2, SCN5A, ADRB2, RXRA, MAOB, CASP9, PLAU, MAPK1 and SOD1, which indicates that these targets also play a relatively important role in the network. They may be the main targets of ephedra-bitter almond to anti-COVID-19.

### PPI analysis

**Fig. 3:**
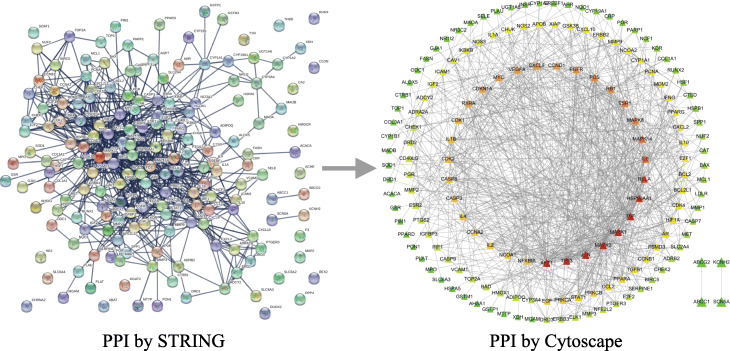
Protein-protein interaction (PPI) network. PPI by Cytoscape: node color represents the size of the degree. The node color was from green to red, and the corresponding degree gradually larger. Node size represents closeness centrality. The node size is proportional to its closeness centrality. Line thickness represents combined score, indicating a closer relationship between the targets

The PPI network was obtained by importing 178 potential targets into the STRING database and setting the species as “*Homo sapiens*”. Subsequently, the PPI network was further visualized and topologically analyzed using Cytoscape software (Fig. 3). The network consists of 159 nodes and 778 edges. It was analyzed by NetworkAnalyzer, the node color was set by the DC value of each node, the size of the node was set by the CC value, and the edge thickness was set according to the BC value of the node. The redder the node color, the larger the DC, and the larger the node size, the larger the CC, indicating that it is more critical in the network; the thicker the edge, the larger the combined score, indicating a closer relationship between the targets. The target nodes have a median DC value of 7, a median CC of 0.3624, and a median BC of 0.0036. Among them, the three centralities of 55 compounds are higher than the median, including AKT1, TP53, JUN, MAPK1, MAPK3, TNF, HSP90AA1, RELA, IL6, MAPK14, ESR1, MAPK8, RB1, FOS, EGFR, CXCL8, VEGFA, CCND1, MYC, RXRA, CDKN1A, CDK1, CASP3, CASP8, IL1B, CDK2, IL4, PRKCA, EGF, NCOA1, IL2, CCNA2, NFKBIA, STAT1, PRKCB, PPARA, CCL2, PSMD3, TGFB1, AR, HIF1A, BCL2, BCL2L1, PPARG, PCNA, IFNG, MMP9, NCOA2, APOB, NOS2, XIAP, IGF2, CAV1, IGFBP3 and PTGS2, which indicates that these targets also play a relatively important role in the PPI network.

The 55 main targets based on the PPI network analysis were compared with the 31 main targets based on the H-C-T network analysis. It is found that 13 targets play an important role in both H-C-T network and PPI network, including PTGS2, HSP90AA1, AR, PPARG, NCOA2, RELA, AKT1, BCL2, TNF, JUN, CASP3, RXRA and MAPK1. These targets not only play a significant role in the anti-COVID-19 process of ephedra-bitter almond but also play a key role in the gene regulatory network. Therefore, they are considered to be the core targets of active component anti-COVID-19.

### Functional annotations

#### GO enrichment analysis

**Fig. 4:**
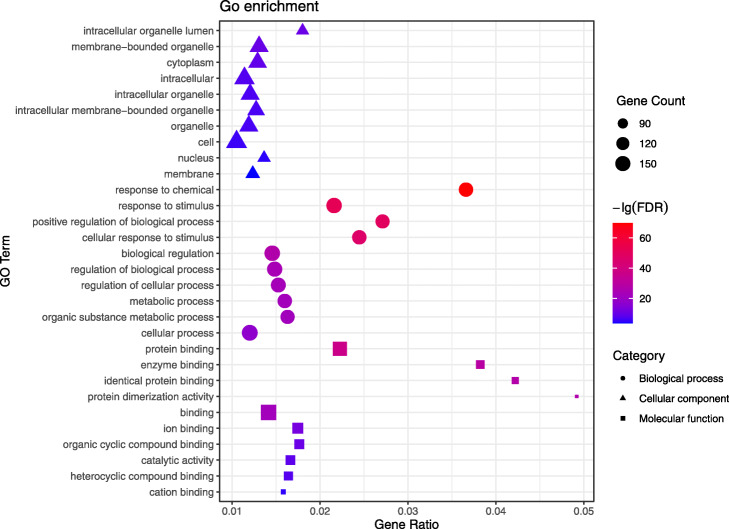
Cellular component, biological process, and molecular function analysis of 179 potential targets. The horizontal axis (Gene Radio) of the bubble diagram represents the ratio of the core targets involved in each term to the total number of targets in the term; the size of the bubble represents the number of core targets involved in the term; and the color from red to blue indicates the *FDR* value from small to large, that is, the redder it is, the higher the significance of the term

GO annotation analysis includes three parts: cellular component, biological process, and molecular function. We analyzed the top 10 GO terms that were the most significant, respectively (See Fig. 4 and Supplementary Material Table S3).

From cellular component ontology analysis, it was significantly correlated with the intracellular organelle lumen (GO: 0070013), membrane-bounded organelle (GO: 0043227), and cytoplasm (GO: 0005737) in cellular components, indicating that the active components of ephedra-bitter almond anti-COVID-19 interact primarily with related targets in the cytoplasm and organelles.

From biological process ontology analysis, the active ingredients of ephedra-bitter almond are mainly through the response to chemical (GO: 0042221), response to stimulus (GO: 0050896), positive regulation of biological process (GO: 0048518), and cellular response to stimulus (GO: 0051716) to against COVID-19. These processes are mainly related to the changes in the state or activity of cells or organisms caused by stimulation, which is consistent with the stimulation of the body after the virus infects the human.

From molecular function ontology analysis, ephedra-bitter almond anti-COVID-19 was mainly associated with functions such as protein binding (GO: 0005515), enzyme binding (GO: 0019899), identical protein binding (GO: 0042802), and protein dimerization activity (GO: 0046983). These molecular functions were primarily involved in the selective interaction with proteins and enzymes, thereby affecting the physiological and biochemical processes of the body.

#### KEGG pathway enrichment analysis

**Fig. 5:**
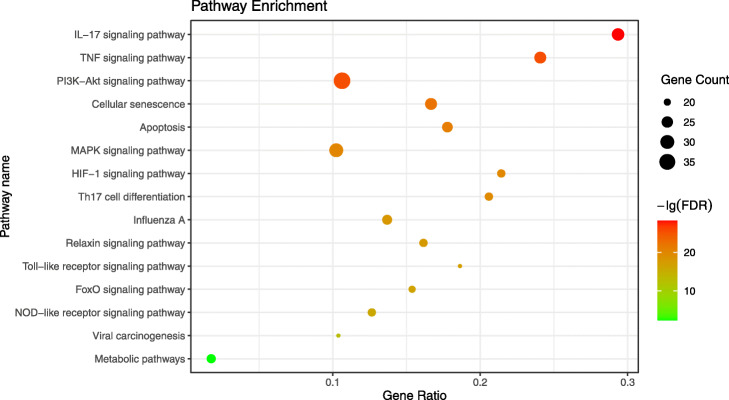
KEGG pathway enrichment analysis of 178 potential targets. The horizontal axis (Gene Radio) of the bubble diagram represents the ratio of the core targets involved in each pathway to the total number of targets in the pathway; the size of the bubble represents the number of core targets involved in the pathway; and the color from red to green indicates the *FDR* value was from small to large, that is, the redder it is, the higher the significance of the pathway

The result showed that the 178 potential targets were mapped to a total of 197 pathways. Next, we excluded pathways unrelated to COVID-19, such as “prostate cancer”, “pancreatic cancer”, “Chagas disease”, identified 58 pathways, and selected the top 15 pathways with the highest number of observed genes for analysis (See Fig. 5 and Supplementary Material Table S4). Through the bubble diagram, it was not difficult to find that the IL-17 signaling pathway (hsa04657), TNF signaling pathway (hsa04668), and PI3K-Akt signaling pathway (hsa04151) were more important pathways.

### Docking results

**Fig. 6:**
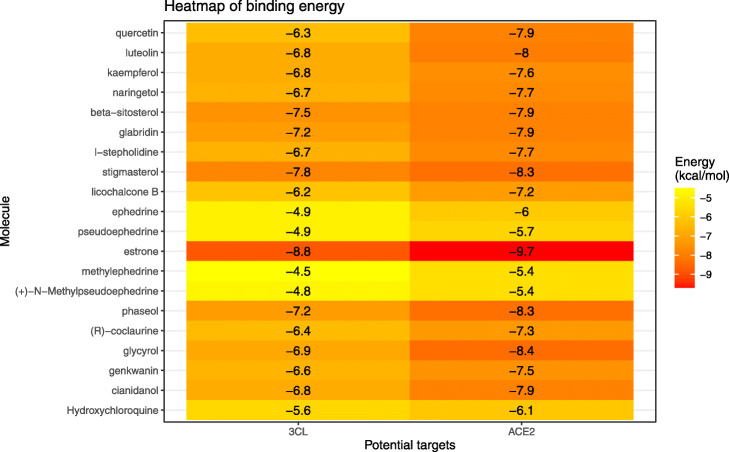
Binding energies heatmap of 19 main compounds and positive control drug to two potential targets. The color from yellow to red indicates the binding energy was from small to large

**Fig. 7:**
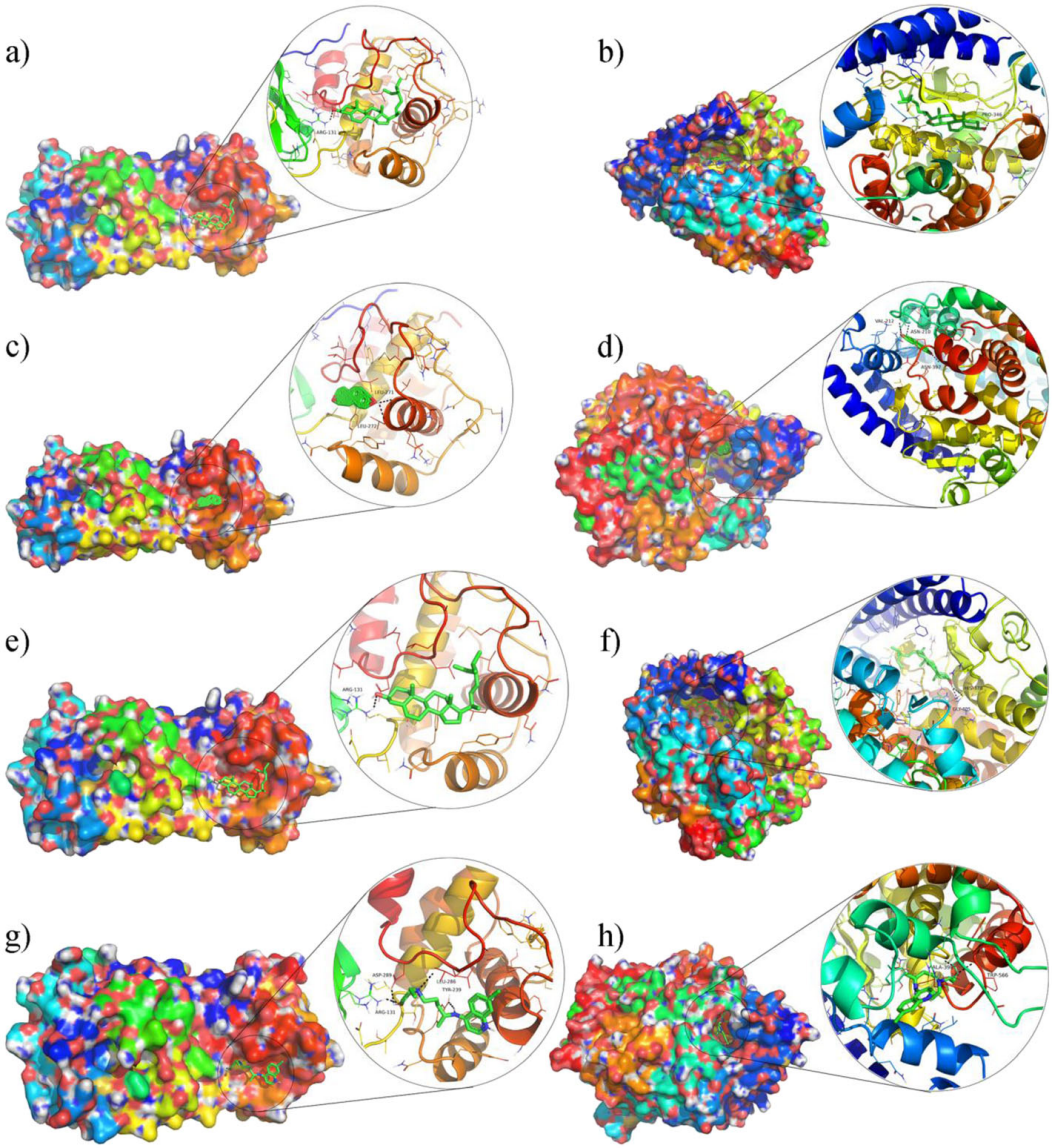
The docking complex of two targets and four components. Colored irregular clumps represent proteins, green chemical structures represent compounds, and each picture shows the details of the docking part. **a** β-sitosterol-3CL, (**b**) β-sitosterol-ACE2; (**c**) estrone-3CL, (**d**) estrone-ACE2, (**e**) stigmasterol-3CL, (**f**) stigmasterol-ACE2, (**g**) Hydroxychloroquine-3CL, (**h**) Hydroxychloroquine-ACE2

We simulated the docking of 19 main compounds of ephedra-bitter almond with two potential targets, 3CL and ACE2, respectively. The binding energies are shown in Table 3 and Fig. 6. Compared with the positive control drug hydroxychloroquine, the binding energies of these active components with 3CL and ACE2 are generally ideal. This further suggests that ephedra-bitter almond anti-COVID-19 may be performed through a multi-component-target-pathway mode. Meanwhile, we also drew the docking patterns of three compounds with higher binding energy and hydroxychloroquine with target proteins (Fig. 7). The prediction of docking patterns and binding residues could provide an important basis for further exploration of drug targets.

**Table 3:** Binding energies of 19 main compounds and positive control drug to two potential targets

No.	Mol ID	Molecule Name	3CL (kcal/mol)	ACE2 (kcal/mol)
1	MOL000098	quercetin	−6.3	−7.9
2	MOL000006	luteolin	−6.8	−8
3	MOL000422	kaempferol	−6.8	−7.6
4	MOL004328	naringetol	−6.7	−7.7
5	MOL000358	β-sitosterol	−7.5	−7.9
6	MOL004908	glabridin	−7.2	−7.9
7	MOL012922	l-stepholidine	−6.7	−7.7
8	MOL000449	stigmasterol	−7.8	−8.3
9	MOL004841	licochalcone B	−6.2	−7.2
10	MOL006594	ephedrine	−4.9	−6
11	MOL006637	pseudoephedrine	−4.9	−5.7
12	MOL010921	estrone	−8.8	−9.7
13	MOL009189	methylephedrine	−4.5	−5.4
14	MOL009194	(+)-N-Methylpseudoephedrine	−4.8	−5.4
15	MOL005017	phaseol	−7.2	−8.3
16	MOL007207	(R)-coclaurine	−6.4	−7.3
17	MOL002311	glycyrol	−6.9	−8.4
18	MOL005573	genkwanin	−6.6	−7.5
19	MOL000492	cianidanol	−6.8	−7.9
20	Positive control	Hydroxychloroquine	−5.6	−6.1

## Discussion

The rapid spread of COVID-19 has alarmed many people [42]. The disease is characterized by fulminated onset and develops into respiratory failure [43]. With no wonder drugs for SARS-CoV-2, some people are turning to TCM, often on the advice of their doctors [44]. TCM drugs have proven to be effective in the treatment of COVID-19, especially for mild and general cases. They have effectively relieved symptoms, cut the rate of patients developing severe conditions, reduced the mortality rate, and boosted patients’ recovery [2]. Nonetheless, no single method is ever going to be universally applicable. Hence, the goal of management is to achieve optimal symptom control.

Ephedra-bitter almond is a common couplet medicine in classic TCM prescriptions for the treatment of upper respiratory tract infections. For instance, Mahuang decoction and Ma Xing Shi Gan decoction both contain ephedra-bitter almond. In this study, network pharmacology combined with molecular docking techniques was used to explore the active components, key targets, and related pathways of ephedra-bitter almond against COVID-19. Recently, some similar studies have also been carried on the beneficial exploration of TCM against COVID-19. For example, Yu-Liang Zhang et al. [45] studied the mechanism of action of Xuebijing injection in the treatment of COVID-19 based on network pharmacology, revealing that this Chinese medicine injection may alleviate the symptoms of COVID-19 by affecting angiotensin-converting enzyme 2 and some key pathways. Compared with the reference, the advantage of our study is that the collection of prescription ingredients is not limited to the TCMSP database, as we also combined with literature mining to supplement the potential active ingredients. Moreover, in order to collect disease targets more comprehensively, we have collected and integrated COVID-19-related targets in the GeneCards and CTD databases respectively, which seems to be more credible and rigorous compared with the method of expanding the collection of targets by STRING tool. Next, we used all the potential targets of the herbs anti-COVID-19 for functional annotation, which explained the potential biological process of herbal treatment of diseases more comprehensively than using key targets alone. We also simultaneously constructed the H-C-T network and PPI network to collect the key targets, taking into account not only the process of prescription treatment of diseases but also the interaction between target genes. Through the study of ephedra-bitter almond, we can initially understand its pharmacodynamic material basis and molecular mechanism of action, thus providing a certain theoretical basis for the development and clinical application of new drugs.

We found that some important components in ephedra-bitter almond, such as quercetin, luteolin, kaempferol, naringetol, β-sitosterol, and glabridin, may play a key role in the prevention and treatment of COVID-19. Here, quercetin, as one of the components with the highest degree value, had certain preventive or therapeutic effects on murine coronavirus, enterovirus 71, human immunodeficiency virus type 1, and dengue virus infection [46–49]. And luteolin was also resistant to dengue virus, influenza A virus, Japanese encephalitis virus, and so on [50–52]. In addition, ephedrine alkaloids, such as ephedrine, pseudoephedrine, and methylephedrine, had potential therapeutic effects on viral-induced respiratory infections [53]. This indicated that ephedra-bitter almond may resist COVID-19 through antiviral and sympathomimetic effects.

H-C-T network and PPI network analysis showed that the active components of ephedra-bitter almond were anti-COVID-19 mainly by regulating PTGS2, HSP90AA1, AR, PPARG, NCOA2, RELA, AKT1, BCL2, TNF, JUN, CASP3, RXRA and MAPK1. Here, PTGS2, also known as cyclooxygenase 2 (COX-2), is a key enzyme in prostaglandin biosynthesis. It was regulated by specific stimulating events and was responsible for the biosynthesis of prostaglandins in the process of inflammation [54]. COX-2 also was regarded as playing an important role in the pathogenesis of airway inflammation in respiratory diseases. Therefore, ephedra-bitter almond may regulate the expression of PTGS2 in the process of anti-COVID-19 and thus treat respiratory inflammation [55]. When pathogens invade cells, autophagy can be activated as an innate immune mechanism to control infection [56]. And there is a highly complex interplay between autophagy and invading viruses. As a highly conserved molecular chaperone, HSP90AA1 may initiate natural cellular defense against invading pathogens [57]. Therefore, ephedra-bitter almond may be helpful against COVID-19 by enhancing the destructive aspects of autophagy on the life cycle of the virus. In the latest network pharmacology research, the mechanism of Qingfei Paidu Decoction and Ma Xing Shi Gan Decoction in the treatment of COVID-19 was studied. It was found that these TCM prescriptions could be anti-COVID-19 through anti-viral, anti-inflammatory activity, and metabolic processes, of which the regulation of immune function may be the main channel [58–60]. These TCM prescriptions all contained ephedra-bitter almond, and the research results were basically consistent with this study.

Functional enrichment analysis showed that ephedra-bitter almond may play a role in anti-COVID-19 by regulating different biological processes and signaling pathways. Virus-infected host cells act as an important immune niche during viral infection and replication, and they stimulate the host’s immune response through molecular signaling [61]. However, as the virus continues to mutate, the body sometimes cannot respond as quickly as needed. At this time, the intervention of drugs or vaccines may increase the body’s sensitivity to viral stimulation, thereby restoring the balance of the immune ecosystem in the infected host tissue [62]. As shown by the results of the GO analysis in this study, ephedra-bitter almond may participate in biological processes such as immune regulation by enhancing the body’s response to pathogen stimulation. As one of the most significant signaling pathways, the PI3K-Akt signaling pathway was involved in the regulation of various cellular functions such as proliferation, differentiation, apoptosis, and glucose transport [63]. It had been reported that the nucleocapsid protein of SARS-CoV may promote the phosphorylation of Akt and JNK in host cells, and the PI3K-Akt pathway played a key role in avoiding apoptosis in SARS-CoV-infected cells [64, 65]. Therefore, the intervention of ephedra-bitter almond on SARS-CoV-2 infected cells may also be carried out through the PI3K-Akt pathway. The excessive inflammatory response in the process of pathogen infection is destructive to the host, and when the production of pro-inflammatory cytokines increases, it causes serious damage to the lungs [66]. A study showed that IL-17 produced during viral infection specially enhanced the pro-inflammatory response by directly cooperating with antiviral signaling [67]. Therefore, the IL-17 signaling pathway played a key role in regulating the immune pathophysiology of viral infection. In addition, COVID-19 can cause a strong immune response and inflammatory storm [68]. And, the inflammatory process extensively mediated by the TNF signaling pathway also had a certain regulatory effect on the occurrence and development of infectious diseases [69, 70]. Also, many studies have confirmed that the active ingredients in ephedra-bitter almond have a regulatory effect on these pathways. For example, ephedrine can reduce the secretion of proinflammatory cytokines through the PI3K-Akt pathway to inhibit the inflammation induced by peptidoglycan [71]. Amygdalin can relieve the symptoms of acute lung injury by inhibiting the production of TNF-α [72]. Luteolin could inhibit inflammatory response via inactivation of the PI3K-Akt pathway in LPS-stimulated RAW 264.7 cells [73]. In conclusion, ephedra-bitter almond may act against COVID-19 mainly through the PI3K-Akt signaling pathway, IL-17 signaling pathway, and TNF signaling pathway.

ACE2 is widely distributed, and is not only a necessary receptor for the invasion of coronaviruses such as SARS-CoV-2, but also a key substance leading to organ damage [74]. Therefore, the search for possible treatment strategies from ACE2 has broad application prospects and clinical value. 3CLpro, as a major protease encoded by the viral genome, is also one of the most attractive drug targets, because it plays a key role in the cleavage of viral polyproteins into functional proteins. Therefore, inhibition of this enzyme is also an effective strategy to block virus replication [75]. In this study, the results of molecular docking indicated that some key components of ephedra-bitter almond, such as β-sitosterol, estrone, and stigmasterol, had higher binding activities to the potential targets of anti-COVID-19. These natural small molecules may play the role of anti-inflammation or direct inhibition of virus replication by regulating 3CL and ACE2 [76, 77], so it is speculated that ephedra-bitter almond could play an anti-COVID-19 role by regulating 3CL and ACE2. This work provides the possibility to discover or design and synthesize effective protease inhibitors as antivirals for COVID-19.

## Conclusion

In summary, ephedra-bitter almonds were used to prevent and treat COVID-19, not only by directly inhibiting the virus but also by regulating immune responses and promoting body repair. However, this work is a prospective study based on data mining, and the findings need to be interpreted with caution. This study can provide a certain theoretical basis for subsequent experiments.

## Supplementary Information

**Additional file 1: Table S1.** Basic information and network topology parameter values of 47 potentially active compounds obtained by ADME screening. **Table S2.** The information and network topology parameter values of 178 potential targets of ephedra-bitter almond against COVID-19. **Table S3.** GO enrichment analysis. **Table S4.** KEGG enrichment analysis.

## Data Availability

All data are available in the manuscript and they are shown in figures and tables.

## Abbreviations

ACE2: Angiotensin-converting enzyme 2,
BC: Betweenness centrality
CC: Closeness centrality
CTD database: Comparative Toxicogenomics Database
COVID-19: Corona Virus Disease 2019
DC: Degree centrality
DL: Drug-likeness
FDR: False Discovery Rate
GO: Gene Ontology
H-C-T: Herb-component-target
KEGG: Kyoto encyclopedia of genes and genomes
OB: Oral bioavailability
PPI: Protein-protein interaction
STRING: Search Tool for Retrieval of Interacting Genes/Proteins
SARS-CoV-2: Severe acute respiratory syndrome coronavirus 2
TCM: Traditional Chinese Medicine
TCMSP Database: Traditional Chinese Medicine Systems Pharmacology Database and Analysis Platform
UniProt database: The Universal Protein Resource
