# Supplementary material for: Exploring active ingredients and function mechanisms of Ephedra-bitter almond for prevention and treatment of Corona virus disease 2019 (COVID-19) based on network pharmacology
Source: BioData Min. 2020 Nov 10;13:19. doi: 10.1186/s13040-020-00229-4 (PMC7653455; doi:10.1186/s13040-020-00229-4)
Supplement: Supplementary file 1 — Additional file 1: Table S1. Basic information and network topology parameter values of 47 potentially active compounds obtained by ADME screening. Table S2. The information and network topology parameter values of 178 potential targets of ephedra-bitter almond against COVID-19. Table S3. GO enrichment analysis. Table S4. KEGG enrichment analysis. [file 13040_2020_229_MOESM1_ESM.docx]

**Supplementary Material**

**Table S1. Basic information and network topology parameter values of 47 potentially active compounds obtained by ADME screening.**

| No. | Mol ID | Molecule Name | OB (%) | DL | DC | CC | BC | Herb |
| --- | --- | --- | --- | --- | --- | --- | --- | --- |
| 1 | MOL000006 | luteolin | 36.16 | 0.25 | 52 | 0.4185 | 0.1497 | Ephedra |
| 2 | MOL000098 | quercetin | 46.43 | 0.28 | 123 | 0.5678 | 0.5875 | Ephedra |
| 3 | MOL000358 | β-sitosterol | 36.91 | 0.75 | 21 | 0.3767 | 0.0342 | Ephedra |
| 4 | MOL000422 | kaempferol | 41.88 | 0.24 | 42 | 0.4050 | 0.0817 | Ephedra |
| 5 | MOL001494 | ethyl linoleate | 42 | 0.19 | 3 | 0.3542 | 0.0002 | Ephedra |
| 6 | MOL001506 | squalene | 33.55 | 0.42 | 1 | 0.3304 | 0.0000 | Ephedra |
| 7 | MOL001755 | Stigmast-4-en-3-one | 36.08 | 0.76 | 3 | 0.3373 | 0.0014 | Ephedra |
| 8 | MOL001771 | clionasterol | 36.91 | 0.75 | 3 | 0.3477 | 0.0005 | Ephedra |
| 9 | MOL002823 | herbacetin | 36.07 | 0.27 | 7 | 0.3542 | 0.0010 | Ephedra |
| 10 | MOL002881 | diosmetin | 31.14 | 0.27 | 7 | 0.3587 | 0.0026 | Ephedra |
| 11 | MOL004328 | naringetol | 59.29 | 0.21 | 29 | 0.3805 | 0.1345 | Ephedra |
| 12 | MOL004576 | taxifolin | 57.84 | 0.27 | 9 | 0.3565 | 0.0164 | Ephedra |
| 13 | MOL004798 | delphinidin | 40.63 | 0.28 | 7 | 0.3587 | 0.0035 | Ephedra |
| 14 | MOL005043 | campesterol | 37.58 | 0.71 | 2 | 0.3363 | 0.0003 | Ephedra |
| 15 | MOL005190 | eriodictyol | 71.79 | 0.24 | 7 | 0.3587 | 0.0033 | Ephedra |
| 16 | MOL005573 | genkwanin | 37.13 | 0.24 | 11 | 0.3633 | 0.0091 | Ephedra |
| 17 | MOL005842 | pectolinarigenin | 41.17 | 0.3 | 8 | 0.3599 | 0.0029 | Ephedra |
| 18 | MOL006594 | ephedrine | 43.35 | 0.03 | 14 | 0.3622 | 0.0082 | Ephedra |
| 19 | MOL006637 | pseudoephedrine | 52.25 | 0.03 | 14 | 0.3622 | 0.0082 | Ephedra |
| 20 | MOL007214 | (+)-leucocyanidin | 37.61 | 0.27 | 3 | 0.3498 | 0.0001 | Ephedra |
| 21 | MOL009189 | methylephedrine | 44.08 | 0.04 | 13 | 0.3610 | 0.0080 | Ephedra |
| 22 | MOL009190 | norephedrine | 66.05 | 0.03 | 7 | 0.3488 | 0.0021 | Ephedra |
| 23 | MOL009191 | norpseudoephedrine | 68.94 | 0.03 | 7 | 0.3488 | 0.0021 | Ephedra |
| 24 | MOL009194 | (+)-N-Methylpseudoephedrine | 37.12 | 0.04 | 13 | 0.3610 | 0.0080 | Ephedra |
| 25 | MOL010489 | leucocianidol | 30.84 | 0.27 | 3 | 0.3498 | 0.0001 | Ephedra |
| 26 | MOL010788 | leucopelargonidin | 57.97 | 0.24 | 4 | 0.3509 | 0.0008 | Ephedra |
| 27 | MOL011319 | Butyl octyl phthalate | 43.74 | 0.24 | 5 | 0.3466 | 0.0007 | Ephedra |
| 28 | MOL000449 | stigmasterol | 43.83 | 0.76 | 17 | 0.3779 | 0.0338 | Ephedra, Bitter almond |
| 29 | MOL000492 | cianidanol | 54.83 | 0.24 | 14 | 0.3669 | 0.0102 | Ephedra, Bitter almond |
| 30 | MOL000211 | betulinic acid | 55.38 | 0.78 | 2 | 0.2586 | 0.0003 | Bitter almond |
| 31 | MOL000359 | sitosterol | 36.91 | 0.75 | 2 | 0.2349 | 0.0088 | Bitter almond |
| 32 | MOL000953 | cholesterol | 37.87 | 0.68 | 4 | 0.3304 | 0.0022 | Bitter almond |
| 33 | MOL001320 | amygdalin | 4.42 | 0.61 | 4 | 0.3553 | 0.0029 | Bitter almond |
| 34 | MOL002211 | 11,14-eicosadienoic acid | 39.99 | 0.2 | 2 | 0.3266 | 0.0007 | Bitter almond |
| 35 | MOL002311 | glycyrol | 90.78 | 0.67 | 11 | 0.3622 | 0.0113 | Bitter almond |
| 36 | MOL002372 | (E,E,E,E)-Squalene | 33.55 | 0.42 | 1 | 0.2344 | 0.0000 | Bitter almond |
| 37 | MOL003410 | Ziziphin_qt | 66.95 | 0.62 | 1 | 0.2344 | 0.0000 | Bitter almond |
| 38 | MOL004355 | α-spinasterol | 42.98 | 0.76 | 4 | 0.3304 | 0.0022 | Bitter almond |
| 39 | MOL004841 | licochalcone B | 76.76 | 0.19 | 16 | 0.3717 | 0.0250 | Bitter almond |
| 40 | MOL004903 | liquiritin | 65.69 | 0.74 | 4 | 0.3542 | 0.0042 | Bitter almond |
| 41 | MOL004908 | glabridin | 53.25 | 0.47 | 19 | 0.3767 | 0.0341 | Bitter almond |
| 42 | MOL005017 | phaseol | 78.77 | 0.58 | 13 | 0.3645 | 0.0199 | Bitter almond |
| 43 | MOL005030 | 11-Eicosenoic acid | 30.7 | 0.2 | 2 | 0.3266 | 0.0007 | Bitter almond |
| 44 | MOL007207 | (R)-coclaurine | 79.64 | 0.24 | 10 | 0.3657 | 0.0085 | Bitter almond |
| 45 | MOL010921 | estrone | 53.56 | 0.32 | 12 | 0.3705 | 0.0182 | Bitter almond |
| 46 | MOL010922 | butanedioic acid | 31.62 | 0.23 | 1 | 0.2344 | 0.0000 | Bitter almond |
| 47 | MOL012922 | l-Stepholidine | 87.35 | 0.54 | 12 | 0.3669 | 0.0197 | Bitter almond |

OB (oral bioavailability), DL (drug-likeness), DC (degree centrality), CC (closeness centrality), BC (betweenness centrality).

**Table S2. The information and network topology parameter values of 178 potential targets of ephedra-bitter almond against COVID-19.**

| No. | UniProt ID | Gene Symbol | Target name | DC | CC | BC |
| --- | --- | --- | --- | --- | --- | --- |
| 1 | P80404 | ABAT | 4-aminobutyrate aminotransferase, mitochondrial | 1 | 0.2759 | 0.0000 |
| 2 | P33527 | ABCC1 | Multidrug resistance-associated protein 1 | 1 | 0.2759 | 0.0000 |
| 3 | Q9UNQ0 | ABCG2 | ATP-binding cassette sub-family G member 2 | 1 | 0.3628 | 0.0000 |
| 4 | Q13085 | ACACA | Acetyl-CoA carboxylase 1 | 1 | 0.3628 | 0.0000 |
| 5 | P22303 | ACHE | Acetylcholinesterase | 10 | 0.4132 | 0.0124 |
| 6 | Q08462 | ADCY2 | Adenylate cyclase type 2 | 1 | 0.2954 | 0.0000 |
| 7 | Q15848 | ADIPOQ | Adiponectin | 1 | 0.2759 | 0.0000 |
| 8 | P08913 | ADRA2A | Alpha-2A adrenergic receptor | 8 | 0.2843 | 0.0007 |
| 9 | P07550 | ADRB2 | Beta-2 adrenergic receptor | 15 | 0.4240 | 0.0269 |
| 10 | P35869 | AHR | Aryl hydrocarbon receptor | 2 | 0.3711 | 0.0004 |
| 11 | O95433 | AHSA1 | Activator of 90 kDa heat shock protein ATPase homolog 1 | 2 | 0.3711 | 0.0004 |
| 12 | P31749 | AKT1 | RAC-alpha serine/threonine-protein kinase | 4 | 0.4117 | 0.0088 |
| 13 | P09917 | ALOX5 | Arachidonate 5-lipoxygenase | 2 | 0.3711 | 0.0004 |
| 14 | P04114 | APOB | Apolipoprotein B-100 | 2 | 0.2787 | 0.0003 |
| 15 | P10275 | AR | Androgen receptor | 9 | 0.4193 | 0.0171 |
| 16 | Q92934 | BAD | Bcl2 antagonist of cell death | 1 | 0.2759 | 0.0000 |
| 17 | Q07812 | BAX | Apoptosis regulator BAX | 3 | 0.3773 | 0.0013 |
| 18 | P10415 | BCL2 | Apoptosis regulator Bcl-2 | 4 | 0.4014 | 0.0075 |
| 19 | Q07817 | BCL2L1 | Bcl-2-like protein 1 | 2 | 0.3773 | 0.0008 |
| 20 | Q9BXY8 | BEX2 | Breast cancer | 1 | 0.1904 | 0.0000 |
| 21 | O15392 | BIRC5 | Baculoviral IAP repeat-containing protein 5 | 2 | 0.3773 | 0.0008 |
| 22 | P00918 | CA2 | Carbonic anhydrase II | 4 | 0.2850 | 0.0004 |
| 23 | P42574 | CASP3 | Caspase-3 | 5 | 0.4193 | 0.0109 |
| 24 | P55210 | CASP7 | Caspase-7 | 1 | 0.2954 | 0.0000 |
| 25 | Q14790 | CASP8 | Caspase-8 | 2 | 0.3711 | 0.0007 |
| 26 | P55211 | CASP9 | Caspase-9 | 3 | 0.3863 | 0.0020 |
| 27 | P04040 | CAT | Catalase | 3 | 0.2857 | 0.0004 |
| 28 | Q03135 | CAV1 | Caveolin-1 | 1 | 0.3628 | 0.0000 |
| 29 | P13500 | CCL2 | C-C motif chemokine 2 | 1 | 0.3628 | 0.0000 |
| 30 | P20248 | CCNA2 | Cyclin-A2 | 4 | 0.2780 | 0.0001 |
| 31 | P14635 | CCNB1 | G2/mitotic-specific cyclin-B1 | 2 | 0.3773 | 0.0008 |
| 32 | P24385 | CCND1 | G1/S-specific cyclin-D1 | 2 | 0.3773 | 0.0008 |
| 33 | P29965 | CD40LG | CD40 ligand | 2 | 0.3773 | 0.0008 |
| 34 | P06493 | CDK1 | Cell division control protein 2 homolog | 2 | 0.3711 | 0.0004 |
| 35 | P24941 | CDK2 | Cell division protein kinase 2 | 3 | 0.2773 | 0.0000 |
| 36 | P11802 | CDK4 | Cell division protein kinase 4 | 1 | 0.2954 | 0.0000 |
| 37 | P38936 | CDKN1A | Cyclin-dependent kinase inhibitor 1 | 2 | 0.3773 | 0.0008 |
| 38 | O14757 | CHEK1 | Serine/threonine-protein kinase Chk1 | 5 | 0.2821 | 0.0003 |
| 39 | O96017 | CHEK2 | Serine/threonine-protein kinase Chk2 | 1 | 0.3628 | 0.0000 |
| 40 | Q15822 | CHRNA2 | Neuronal acetylcholine receptor subunit alpha-2 | 3 | 0.2794 | 0.0001 |
| 41 | O15111 | CHUK | Inhibitor of nuclear factor kappa-B kinase subunit alpha | 1 | 0.3628 | 0.0000 |
| 42 | O14493 | CLDN4 | Claudin-4 | 1 | 0.3628 | 0.0000 |
| 43 | P02452 | COL1A1 | Collagen alpha-1(I) chain | 1 | 0.3628 | 0.0000 |
| 44 | P02461 | COL3A1 | Collagen alpha-1(III) chain | 1 | 0.3628 | 0.0000 |
| 45 | P02741 | CRP | C-reactive protein | 1 | 0.3628 | 0.0000 |
| 46 | P17538 | CTRB1 | Chymotrypsinogen B | 1 | 0.2746 | 0.0000 |
| 47 | P07339 | CTSD | Cathepsin D | 1 | 0.3628 | 0.0000 |
| 48 | P02778 | CXCL10 | C-X-C motif chemokine 10 | 1 | 0.3628 | 0.0000 |
| 49 | P19875 | CXCL2 | C-X-C motif chemokine 2 | 1 | 0.3628 | 0.0000 |
| 50 | P10145 | CXCL8 | Interleukin-8 | 1 | 0.3628 | 0.0000 |
| 51 | P11511 | CYP19A1 | Cytochrome P450 19A1 | 1 | 0.2759 | 0.0000 |
| 52 | P04798 | CYP1A1 | Cytochrome P450 1A1 | 2 | 0.3711 | 0.0004 |
| 53 | P05177 | CYP1A2 | Cytochrome P450 1A2 | 2 | 0.3711 | 0.0004 |
| 54 | Q16678 | CYP1B1 | Cytochrome P450 1B1 | 2 | 0.3711 | 0.0004 |
| 55 | P08684 | CYP3A4 | Cytochrome P450 3A4 | 2 | 0.3711 | 0.0004 |
| 56 | Q96PD7 | DGAT2 | Diacylglycerol O-acyltransferase 2 | 1 | 0.2631 | 0.0000 |
| 57 | P27487 | DPP4 | Dipeptidyl peptidase IV | 13 | 0.4132 | 0.0175 |
| 58 | P21728 | DRD1 | Dopamine D1 receptor | 5 | 0.2879 | 0.0006 |
| 59 | P14416 | DRD2 | D(2) dopamine receptor | 1 | 0.2707 | 0.0000 |
| 60 | P35462 | DRD3 | D(3) dopamine receptor | 1 | 0.2687 | 0.0000 |
| 61 | Q9NRD8 | DUOX2 | Dual oxidase 2 | 1 | 0.3628 | 0.0000 |
| 62 | Q01094 | E2F1 | Transcription factor E2F1 | 1 | 0.3628 | 0.0000 |
| 63 | Q14209 | E2F2 | Transcription factor E2F2 | 1 | 0.3628 | 0.0000 |
| 64 | P01133 | EGF | Pro-epidermal growth factor | 1 | 0.3628 | 0.0000 |
| 65 | P00533 | EGFR | Epidermal growth factor receptor | 2 | 0.3773 | 0.0008 |
| 66 | P19419 | ELK1 | ETS domain-containing protein Elk-1 | 1 | 0.3628 | 0.0000 |
| 67 | P04626 | ERBB2 | Receptor tyrosine-protein kinase erbB-2 | 2 | 0.3773 | 0.0008 |
| 68 | P21860 | ERBB3 | Receptor tyrosine-protein kinase erbB-3 | 1 | 0.3628 | 0.0000 |
| 69 | P03372 | ESR1 | Estrogen receptor | 8 | 0.3009 | 0.0045 |
| 70 | Q92731 | ESR2 | Estrogen receptor beta | 3 | 0.2800 | 0.0001 |
| 71 | P13726 | F3 | Tissue factor | 1 | 0.3628 | 0.0000 |
| 72 | P49327 | FASN | Fatty acid synthase | 1 | 0.2759 | 0.0000 |
| 73 | P01100 | FOS | Proto-oncogene c-Fos | 1 | 0.3628 | 0.0000 |
| 74 | P17302 | GJA1 | Gap junction alpha-1 protein | 1 | 0.3628 | 0.0000 |
| 75 | P49841 | GSK3B | Glycogen synthase kinase-3 beta | 4 | 0.2780 | 0.0001 |
| 76 | P00390 | GSR | Glutathione reductase, mitochondrial | 1 | 0.2759 | 0.0000 |
| 77 | P09488 | GSTM1 | Glutathione S-transferase Mu 1 | 2 | 0.3711 | 0.0004 |
| 78 | P09211 | GSTP1 | Glutathione S-transferase P | 4 | 0.4117 | 0.0088 |
| 79 | Q16665 | HIF1A | Hypoxia-inducible factor 1-alpha | 1 | 0.3628 | 0.0000 |
| 80 | P52789 | HK2 | Hexokinase-2 | 1 | 0.3628 | 0.0000 |
| 81 | P04035 | HMGCR | 3-hydroxy-3-methylglutaryl-coenzyme A reductase | 1 | 0.2759 | 0.0000 |
| 82 | P09601 | HMOX1 | Heme oxygenase 1 | 4 | 0.3877 | 0.0023 |
| 83 | Q00613 | HSF1 | Heat shock factor protein 1 | 1 | 0.3628 | 0.0000 |
| 84 | P07900 | HSP90AA1 | Heat shock protein HSP 90 | 21 | 0.4819 | 0.0562 |
| 85 | P11021 | HSPA5 | 78 kDa glucose-regulated protein | 1 | 0.3628 | 0.0000 |
| 86 | P04792 | HSPB1 | Heat shock protein beta-1 | 1 | 0.3628 | 0.0000 |
| 87 | P05362 | ICAM1 | Intercellular adhesion molecule 1 | 4 | 0.3917 | 0.0038 |
| 88 | P01579 | IFNG | Interferon gamma | 2 | 0.3773 | 0.0008 |
| 89 | P01344 | IGF2 | Insulin-like growth factor II | 1 | 0.3628 | 0.0000 |
| 90 | P17936 | IGFBP3 | Insulin-like growth factor-binding protein 3 | 1 | 0.3628 | 0.0000 |
| 91 | O14920 | IKBKB | Inhibitor of nuclear factor kappa-B kinase subunit beta | 1 | 0.2886 | 0.0000 |
| 92 | P22301 | IL10 | Interleukin-10 | 2 | 0.3773 | 0.0008 |
| 93 | P01583 | IL1A | Interleukin-1 alpha | 1 | 0.3628 | 0.0000 |
| 94 | P01584 | IL1B | Interleukin-1 beta | 1 | 0.3628 | 0.0000 |
| 95 | P60568 | IL2 | Interleukin-2 | 2 | 0.3773 | 0.0008 |
| 96 | P05112 | IL4 | Interleukin-4 | 1 | 0.2954 | 0.0000 |
| 97 | P05231 | IL6 | Interleukin-6 | 2 | 0.3773 | 0.0008 |
| 98 | P06213 | INSR | Insulin receptor | 3 | 0.3863 | 0.0015 |
| 99 | P10914 | IRF1 | Interferon regulatory factor 1 | 1 | 0.3628 | 0.0000 |
| 100 | P05412 | JUN | Transcription factor AP-1 | 4 | 0.3930 | 0.0029 |
| 101 | Q12809 | KCNH2 | Potassium voltage-gated channel subfamily H member 2 | 3 | 0.3837 | 0.0027 |
| 102 | P35968 | KDR | Vascular endothelial growth factor receptor 2 | 3 | 0.2700 | 0.0001 |
| 103 | P01130 | LDLR | Low-density lipoprotein receptor | 1 | 0.2759 | 0.0000 |
| 104 | P21397 | MAOA | Amine oxidase [flavin-containing] A | 5 | 0.2800 | 0.0003 |
| 105 | P27338 | MAOB | Amine oxidase [flavin-containing] B | 4 | 0.3863 | 0.0034 |
| 106 | P11137 | MAP2 | Microtubule-associated protein 2 | 1 | 0.2739 | 0.0000 |
| 107 | P28482 | MAPK1 | Mitogen-activated protein kinase 1 | 3 | 0.4014 | 0.0070 |
| 108 | Q16539 | MAPK14 | Mitogen-activated protein kinase 14 | 4 | 0.2780 | 0.0001 |
| 109 | P27361 | MAPK3 | Mitogen-activated protein kinase 3 | 1 | 0.2759 | 0.0000 |
| 110 | P45983 | MAPK8 | Mitogen-activated protein kinase 8 | 1 | 0.2886 | 0.0000 |
| 111 | Q07820 | MCL1 | Induced myeloid leukemia cell differentiation protein Mcl-1 | 1 | 0.2954 | 0.0000 |
| 112 | Q00987 | MDM2 | E3 ubiquitin-protein ligase Mdm2 | 1 | 0.2954 | 0.0000 |
| 113 | P08581 | MET | Hepatocyte growth factor receptor | 1 | 0.2954 | 0.0000 |
| 114 | O43451 | MGAM | Maltase-glucoamylase, intestinal | 1 | 0.3628 | 0.0000 |
| 115 | P03956 | MMP1 | Interstitial collagenase | 3 | 0.3863 | 0.0015 |
| 116 | P08253 | MMP2 | 72 kDa type IV collagenase | 2 | 0.3773 | 0.0008 |
| 117 | P08254 | MMP3 | Stromelysin-1 | 1 | 0.3628 | 0.0000 |
| 118 | P14780 | MMP9 | Matrix metalloproteinase-9 | 2 | 0.3773 | 0.0008 |
| 119 | P05164 | MPO | Myeloperoxidase | 1 | 0.3628 | 0.0000 |
| 120 | P55157 | MTTP | Microsomal triglyceride transfer protein large subunit | 2 | 0.2787 | 0.0003 |
| 121 | P01106 | MYC | Myc proto-oncogene protein | 1 | 0.3628 | 0.0000 |
| 122 | P14598 | NCF1 | Neutrophil cytosol factor 1 | 1 | 0.3628 | 0.0000 |
| 123 | Q15788 | NCOA1 | Nuclear receptor coactivator 1 | 5 | 0.2872 | 0.0005 |
| 124 | Q15596 | NCOA2 | Nuclear receptor coactivator 2 | 21 | 0.4529 | 0.0606 |
| 125 | Q16236 | NFE2L2 | Nuclear factor erythroid 2-related factor 2 | 2 | 0.3639 | 0.0005 |
| 126 | P25963 | NFKBIA | NF-kappa-B inhibitor alpha | 2 | 0.3773 | 0.0008 |
| 127 | P35228 | NOS2 | Nitric oxide synthase, inducible | 8 | 0.3100 | 0.0029 |
| 128 | P29474 | NOS3 | Nitric-oxide synthase, endothelial | 6 | 0.3837 | 0.0042 |
| 129 | P15559 | NQO1 | NAD(P)H dehydrogenase [quinone] 1 | 2 | 0.3639 | 0.0005 |
| 130 | O75469 | NR1I2 | Nuclear receptor subfamily 1 group I member 2 | 2 | 0.3711 | 0.0004 |
| 131 | P08235 | NR3C2 | Mineralocorticoid receptor | 4 | 0.2766 | 0.0005 |
| 132 | Q9BZD4 | NUF2 | Kinetochore protein Nuf2 | 1 | 0.2954 | 0.0000 |
| 133 | P11926 | ODC1 | Ornithine decarboxylase | 1 | 0.3628 | 0.0000 |
| 134 | P09874 | PARP1 | Poly [ADP-ribose] polymerase 1 | 1 | 0.3628 | 0.0000 |
| 135 | P12004 | PCNA | Proliferating cell nuclear antigen | 1 | 0.2954 | 0.0000 |
| 136 | P06401 | PGR | Progesterone receptor | 10 | 0.3197 | 0.0117 |
| 137 | P11309 | PIM1 | Proto-oncogene serine/threonine-protein kinase Pim-1 | 4 | 0.2780 | 0.0001 |
| 138 | P00750 | PLAT | Tissue-type plasminogen activator | 1 | 0.3628 | 0.0000 |
| 139 | P00749 | PLAU | Urokinase-type plasminogen activator | 4 | 0.3863 | 0.0034 |
| 140 | P27169 | PON1 | Serum paraoxonase/arylesterase 1 | 2 | 0.3711 | 0.0007 |
| 141 | P16435 | POR | NADPH--cytochrome P450 reductase | 1 | 0.3628 | 0.0000 |
| 142 | Q07869 | PPARA | Peroxisome proliferator-activated receptor alpha | 2 | 0.3850 | 0.0045 |
| 143 | Q03181 | PPARD | Peroxisome proliferator-activated receptor delta | 1 | 0.3628 | 0.0000 |
| 144 | P37231 | PPARG | Peroxisome proliferator activated receptor gamma | 9 | 0.4458 | 0.0299 |
| 145 | P17252 | PRKCA | Protein kinase C alpha type | 2 | 0.3711 | 0.0007 |
| 146 | P05771 | PRKCB | Protein kinase C beta type | 1 | 0.3628 | 0.0000 |
| 147 | O43242 | PSMD3 | 26S proteasome non-ATPase regulatory subunit 3 | 2 | 0.3711 | 0.0004 |
| 148 | P43115 | PTGER3 | Prostaglandin E2 receptor EP3 subtype | 1 | 0.3628 | 0.0000 |
| 149 | P35354 | PTGS2 | Prostaglandin G/H synthase 2 | 32 | 0.5148 | 0.1146 |
| 150 | P06400 | RB1 | Retinoblastoma-associated protein | 2 | 0.3773 | 0.0008 |
| 151 | Q04206 | RELA | Transcription factor p65 | 5 | 0.4147 | 0.0113 |
| 152 | Q06455 | RUNX1T1 | Protein CBFA2T1 | 1 | 0.3628 | 0.0000 |
| 153 | Q13950 | RUNX2 | Runt-related transcription factor 2 | 1 | 0.3628 | 0.0000 |
| 154 | P19793 | RXRA | Retinoic acid receptor RXR-alpha | 10 | 0.4177 | 0.0157 |
| 155 | Q14524 | SCN5A | Sodium channel protein type 5 subunit alpha | 12 | 0.4177 | 0.0162 |
| 156 | P16581 | SELE | E-selectin | 2 | 0.3711 | 0.0004 |
| 157 | P05121 | SERPINE1 | Plasminogen activator inhibitor 1 | 1 | 0.3628 | 0.0000 |
| 158 | P14672 | SLC2A4 | Solute carrier family 2, facilitated glucose transporter member 4 | 3 | 0.3863 | 0.0015 |
| 159 | P23975 | SLC6A2 | Sodium-dependent noradrenaline transporter | 10 | 0.3135 | 0.0035 |
| 160 | Q01959 | SLC6A3 | Sodium-dependent dopamine transporter | 11 | 0.2894 | 0.0021 |
| 161 | P31645 | SLC6A4 | Sodium-dependent serotonin transporter | 8 | 0.2924 | 0.0013 |
| 162 | P35610 | SOAT1 | Sterol O-acyltransferase 1 | 1 | 0.2759 | 0.0000 |
| 163 | P00441 | SOD1 | Superoxide dismutase [Cu-Zn] | 3 | 0.3972 | 0.0076 |
| 164 | P10451 | SPP1 | Osteopontin | 1 | 0.3628 | 0.0000 |
| 165 | P36956 | SREBF1 | Sterol regulatory element-binding protein 1 | 1 | 0.2759 | 0.0000 |
| 166 | P42224 | STAT1 | Signal transducer and activator of transcription 1-alpha/beta | 2 | 0.3711 | 0.0004 |
| 167 | P01137 | TGFB1 | Transforming growth factor beta-1 | 2 | 0.3711 | 0.0007 |
| 168 | P07204 | THBD | Thrombomodulin | 1 | 0.3628 | 0.0000 |
| 169 | P01375 | TNF | Tumor necrosis factor | 3 | 0.3863 | 0.0015 |
| 170 | P11387 | TOP1 | DNA topoisomerase 1 | 2 | 0.3773 | 0.0008 |
| 171 | P11388 | TOP2A | DNA topoisomerase 2-alpha | 2 | 0.3773 | 0.0008 |
| 172 | P04637 | TP53 | Cellular tumor antigen p53 | 2 | 0.3773 | 0.0008 |
| 173 | P14679 | TYR | Tyrosinase | 1 | 0.2954 | 0.0000 |
| 174 | Q9HAW9 | UGT1A8 | UDP-glucuronosyltransferase 1-1 | 1 | 0.2759 | 0.0000 |
| 175 | P19320 | VCAM1 | Vascular cell adhesion protein 1 | 2 | 0.3711 | 0.0004 |
| 176 | P15692 | VEGFA | Vascular endothelial growth factor A | 2 | 0.3773 | 0.0008 |
| 177 | P47989 | XDH | Xanthine dehydrogenase/oxidase | 3 | 0.3863 | 0.0015 |
| 178 | P98170 | XIAP | Baculoviral IAP repeat-containing protein 4 | 1 | 0.2954 | 0.0000 |

DC (degree centrality), CC (closeness centrality), BC (betweenness centrality).

**Table S3.** GO enrichment analysis.

| term ID | term description | observed gene count | background gene count | false discovery rate | Category | matching proteins in your network (labels) |
| --- | --- | --- | --- | --- | --- | --- |
| GO:0070013 | intracellular organelle lumen | 93 | 5162 | 1.77E-11 | Cellular component | ABAT,ACACA,AHR,AKT1,ALOX5,APOB,AR,BCL2,BCL2L1,BIRC5,CASP3,CASP7,CASP8,CAT,CCNA2,CCNB1,CCND1,CDK1,CDK2,CDK4,CDKN1A,CHEK1,CHEK2,CHUK,COL1A1,COL3A1,CTSD,E2F1,E2F2,EGF,EGFR,ELK1,ESR1,ESR2,FOS,GJA1,GSK3B,GSR,GSTP1,HIF1A,HMOX1,HSF1,HSP90AA1,HSPA5,IGF2,IGFBP3,IL6,IRF1,JUN,MAP2,MAPK1,MAPK14,MAPK3,MAPK8,MCL1,MDM2,MMP9,MPO,MTTP,MYC,NCOA1,NCOA2,NFE2L2,NOS2,NR1I2,NR3C2,PARP1,PCNA,PGR,PIM1,PPARA,PPARD,PPARG,PRKCA,PRKCB,PSMD3,PTGS2,RB1,RELA,RUNX1T1,RUNX2,RXRA,SERPINE1,SOD1,SPP1,SREBF1,STAT1,TGFB1,TOP1,TOP2A,TP53,VEGFA,XIAP |
| GO:0043227 | membrane-bounded organelle | 147 | 11244 | 4.02E-11 | Cellular component | ABAT,ABCG2,ACACA,ACHE,ADIPOQ,ADRB2,AHR,AHSA1,AKT1,ALOX5,APOB,AR,BAD,BAX,BCL2,BCL2L1,BEX2,BIRC5,CASP3,CASP7,CASP8,CASP9,CAT,CAV1,CCNA2,CCNB1,CCND1,CDK1,CDK2,CDK4,CDKN1A,CHEK1,CHEK2,CHUK,COL1A1,COL3A1,CTSD,CYP19A1,CYP1A1,CYP1A2,CYP1B1,CYP3A4,DGAT2,DPP4,DRD1,DRD2,DRD3,E2F1,E2F2,EGF,EGFR,ELK1,ERBB2,ESR1,ESR2,FASN,FOS,GJA1,GSK3B,GSR,GSTP1,HIF1A,HK2,HMGCR,HMOX1,HSF1,HSP90AA1,HSPA5,HSPB1,ICAM1,IGF2,IGFBP3,IKBKB,IL1B,IL6,INSR,IRF1,JUN,KDR,LDLR,MAOA,MAOB,MAP2,MAPK1,MAPK14,MAPK3,MAPK8,MCL1,MDM2,MGAM,MMP2,MMP9,MPO,MTTP,MYC,NCF1,NCOA1,NCOA2,NFE2L2,NFKBIA,NOS2,NOS3,NR1I2,NR3C2,NUF2,PARP1,PCNA,PGR,PIM1,PLAT,PLAU,PON1,POR,PPARA,PPARD,PPARG,PRKCA,PRKCB,PSMD3,PTGER3,PTGS2,RB1,RELA,RUNX1T1,RUNX2,RXRA,SCN5A,SERPINE1,SLC2A4,SLC6A4,SOAT1,SOD1,SPP1,SREBF1,STAT1,TGFB1,THBD,TNF,TOP1,TOP2A,TP53,TYR,UGT1A8,VCAM1,VEGFA,XDH,XIAP |
| GO:0005737 | cytoplasm | 145 | 11238 | 3.94E-10 | Cellular component | ABAT,ABCG2,ACACA,ACHE,ADCY2,ADIPOQ,ADRA2A,ADRB2,AHR,AHSA1,AKT1,ALOX5,APOB,AR,BAD,BAX,BCL2,BCL2L1,BEX2,BIRC5,CA2,CASP3,CASP7,CASP8,CASP9,CAT,CAV1,CCNA2,CCNB1,CCND1,CDK1,CDK2,CDK4,CDKN1A,CHEK1,CHEK2,CHUK,COL1A1,COL3A1,CTSD,CYP19A1,CYP1A1,CYP1A2,CYP1B1,CYP3A4,DGAT2,DPP4,DRD1,DRD2,DRD3,E2F1,EGF,EGFR,ELK1,ERBB2,ESR1,ESR2,FASN,FOS,GJA1,GSK3B,GSR,GSTM1,GSTP1,HIF1A,HK2,HMGCR,HMOX1,HSF1,HSP90AA1,HSPA5,HSPB1,IGF2,IGFBP3,IKBKB,IL1A,IL1B,IL6,INSR,IRF1,JUN,KCNH2,KDR,LDLR,MAOA,MAOB,MAP2,MAPK1,MAPK14,MAPK3,MAPK8,MCL1,MDM2,MGAM,MMP2,MMP9,MPO,MTTP,NCF1,NCOA1,NCOA2,NFE2L2,NFKBIA,NOS2,NOS3,NQO1,NR3C2,NUF2,ODC1,PARP1,PGR,PIM1,PLAT,PLAU,POR,PPARG,PRKCA,PRKCB,PSMD3,PTGS2,RELA,RUNX2,SCN5A,SELE,SERPINE1,SLC2A4,SLC6A3,SLC6A4,SOAT1,SOD1,SPP1,SREBF1,STAT1,TGFB1,THBD,TNF,TOP1,TOP2A,TP53,TYR,UGT1A8,VCAM1,VEGFA,XDH,XIAP |
| GO:0005622 | intracellular | 163 | 14286 | 1.82E-08 | Cellular component | ABAT,ABCG2,ACACA,ACHE,ADCY2,ADIPOQ,ADRA2A,ADRB2,AHR,AHSA1,AKT1,ALOX5,APOB,AR,BAD,BAX,BCL2,BCL2L1,BEX2,BIRC5,CA2,CASP3,CASP7,CASP8,CASP9,CAT,CAV1,CCL2,CCNA2,CCNB1,CCND1,CD40LG,CDK1,CDK2,CDK4,CDKN1A,CHEK1,CHEK2,CHUK,COL1A1,COL3A1,CTSD,CXCL8,CYP19A1,CYP1A1,CYP1A2,CYP1B1,CYP3A4,DGAT2,DPP4,DRD1,DRD2,DRD3,E2F1,E2F2,EGF,EGFR,ELK1,ERBB2,ERBB3,ESR1,ESR2,FASN,FOS,GJA1,GSK3B,GSR,GSTM1,GSTP1,HIF1A,HK2,HMGCR,HMOX1,HSF1,HSP90AA1,HSPA5,HSPB1,IFNG,IGF2,IGFBP3,IKBKB,IL1A,IL1B,IL2,IL6,INSR,IRF1,JUN,KCNH2,KDR,LDLR,MAOA,MAOB,MAP2,MAPK1,MAPK14,MAPK3,MAPK8,MCL1,MDM2,MET,MGAM,MMP2,MMP9,MPO,MTTP,MYC,NCF1,NCOA1,NCOA2,NFE2L2,NFKBIA,NOS2,NOS3,NQO1,NR1I2,NR3C2,NUF2,ODC1,PARP1,PCNA,PGR,PIM1,PLAT,PLAU,PON1,POR,PPARA,PPARD,PPARG,PRKCA,PRKCB,PSMD3,PTGER3,PTGS2,RB1,RELA,RUNX1T1,RUNX2,RXRA,SCN5A,SELE,SERPINE1,SLC2A4,SLC6A3,SLC6A4,SOAT1,SOD1,SPP1,SREBF1,STAT1,TGFB1,THBD,TNF,TOP1,TOP2A,TP53,TYR,UGT1A8,VCAM1,VEGFA,XDH,XIAP |
| GO:0043229 | intracellular organelle | 147 | 12193 | 9.27E-08 | Cellular component | ABAT,ABCG2,ACACA,ACHE,ADIPOQ,ADRB2,AHR,AHSA1,AKT1,ALOX5,APOB,AR,BAD,BAX,BCL2,BCL2L1,BEX2,BIRC5,CASP3,CASP7,CASP8,CASP9,CAT,CAV1,CCNA2,CCNB1,CCND1,CDK1,CDK2,CDK4,CDKN1A,CHEK1,CHEK2,CHUK,COL1A1,COL3A1,CTSD,CYP19A1,CYP1A1,CYP1A2,CYP1B1,CYP3A4,DGAT2,DPP4,DRD1,DRD2,DRD3,E2F1,E2F2,EGF,EGFR,ELK1,ERBB2,ESR1,ESR2,FASN,FOS,GJA1,GSK3B,GSR,GSTP1,HIF1A,HK2,HMGCR,HMOX1,HSF1,HSP90AA1,HSPA5,HSPB1,IGF2,IGFBP3,IKBKB,IL1B,IL6,INSR,IRF1,JUN,KDR,LDLR,MAOA,MAOB,MAP2,MAPK1,MAPK14,MAPK3,MAPK8,MCL1,MDM2,MGAM,MMP2,MMP9,MPO,MTTP,MYC,NCF1,NCOA1,NCOA2,NFE2L2,NFKBIA,NOS2,NOS3,NR1I2,NR3C2,NUF2,PARP1,PCNA,PGR,PIM1,PLAT,PLAU,PON1,POR,PPARA,PPARD,PPARG,PRKCA,PRKCB,PSMD3,PTGER3,PTGS2,RB1,RELA,RUNX1T1,RUNX2,RXRA,SCN5A,SELE,SERPINE1,SLC2A4,SLC6A4,SOAT1,SOD1,SPP1,SREBF1,STAT1,TGFB1,THBD,TNF,TOP1,TOP2A,TP53,TYR,UGT1A8,VCAM1,VEGFA,XDH,XIAP |
| GO:0043231 | intracellular membrane-bounded organelle | 132 | 10365 | 1.34E-07 | Cellular component | ABAT,ABCG2,ACACA,ACHE,ADIPOQ,ADRB2,AHR,AHSA1,AKT1,ALOX5,APOB,AR,BAD,BAX,BCL2,BCL2L1,BEX2,BIRC5,CASP3,CASP7,CASP8,CASP9,CAT,CAV1,CCNA2,CCNB1,CCND1,CDK1,CDK2,CDK4,CDKN1A,CHEK1,CHEK2,CHUK,COL1A1,COL3A1,CTSD,CYP19A1,CYP1A1,CYP1A2,CYP1B1,CYP3A4,DGAT2,DRD1,E2F1,E2F2,EGFR,ELK1,ERBB2,ESR1,ESR2,FASN,FOS,GJA1,GSK3B,GSR,GSTP1,HIF1A,HK2,HMGCR,HMOX1,HSF1,HSP90AA1,HSPA5,HSPB1,IGFBP3,IKBKB,IL1B,IL6,IRF1,JUN,KDR,LDLR,MAOA,MAOB,MAP2,MAPK1,MAPK14,MAPK3,MAPK8,MCL1,MDM2,MMP2,MPO,MTTP,MYC,NCF1,NCOA1,NCOA2,NFE2L2,NFKBIA,NOS2,NOS3,NR1I2,NR3C2,NUF2,PARP1,PCNA,PGR,PIM1,PON1,POR,PPARA,PPARD,PPARG,PRKCA,PRKCB,PSMD3,PTGER3,PTGS2,RB1,RELA,RUNX1T1,RUNX2,RXRA,SCN5A,SLC2A4,SOAT1,SOD1,SPP1,SREBF1,STAT1,TGFB1,THBD,TOP1,TOP2A,TP53,TYR,UGT1A8,VCAM1,XDH,XIAP |
| GO:0043226 | organelle | 148 | 12432 | 1.71E-07 | Cellular component | ABAT,ABCG2,ACACA,ACHE,ADIPOQ,ADRB2,AHR,AHSA1,AKT1,ALOX5,APOB,AR,BAD,BAX,BCL2,BCL2L1,BEX2,BIRC5,CASP3,CASP7,CASP8,CASP9,CAT,CAV1,CCNA2,CCNB1,CCND1,CDK1,CDK2,CDK4,CDKN1A,CHEK1,CHEK2,CHUK,COL1A1,COL3A1,CTSD,CYP19A1,CYP1A1,CYP1A2,CYP1B1,CYP3A4,DGAT2,DPP4,DRD1,DRD2,DRD3,E2F1,E2F2,EGF,EGFR,ELK1,ERBB2,ESR1,ESR2,FASN,FOS,GJA1,GSK3B,GSR,GSTP1,HIF1A,HK2,HMGCR,HMOX1,HSF1,HSP90AA1,HSPA5,HSPB1,ICAM1,IGF2,IGFBP3,IKBKB,IL1B,IL6,INSR,IRF1,JUN,KDR,LDLR,MAOA,MAOB,MAP2,MAPK1,MAPK14,MAPK3,MAPK8,MCL1,MDM2,MGAM,MMP2,MMP9,MPO,MTTP,MYC,NCF1,NCOA1,NCOA2,NFE2L2,NFKBIA,NOS2,NOS3,NR1I2,NR3C2,NUF2,PARP1,PCNA,PGR,PIM1,PLAT,PLAU,PON1,POR,PPARA,PPARD,PPARG,PRKCA,PRKCB,PSMD3,PTGER3,PTGS2,RB1,RELA,RUNX1T1,RUNX2,RXRA,SCN5A,SELE,SERPINE1,SLC2A4,SLC6A4,SOAT1,SOD1,SPP1,SREBF1,STAT1,TGFB1,THBD,TNF,TOP1,TOP2A,TP53,TYR,UGT1A8,VCAM1,VEGFA,XDH,XIAP |
| GO:0005623 | cell | 171 | 16271 | 1.37E-06 | Cellular component | ABAT,ABCC1,ABCG2,ACACA,ACHE,ADCY2,ADIPOQ,ADRA2A,ADRB2,AHR,AHSA1,AKT1,ALOX5,APOB,AR,BAD,BAX,BCL2,BCL2L1,BEX2,BIRC5,CA2,CASP3,CASP7,CASP8,CASP9,CAT,CAV1,CCL2,CCNA2,CCNB1,CCND1,CD40LG,CDK1,CDK2,CDK4,CDKN1A,CHEK1,CHEK2,CHRNA2,CHUK,CLDN4,COL1A1,COL3A1,CTSD,CXCL10,CXCL8,CYP19A1,CYP1A1,CYP1A2,CYP1B1,CYP3A4,DGAT2,DPP4,DRD1,DRD2,DRD3,DUOX2,E2F1,E2F2,EGF,EGFR,ELK1,ERBB2,ERBB3,ESR1,ESR2,F3,FASN,FOS,GJA1,GSK3B,GSR,GSTM1,GSTP1,HIF1A,HK2,HMGCR,HMOX1,HSF1,HSP90AA1,HSPA5,HSPB1,ICAM1,IFNG,IGF2,IGFBP3,IKBKB,IL1A,IL1B,IL2,IL6,INSR,IRF1,JUN,KCNH2,KDR,LDLR,MAOA,MAOB,MAP2,MAPK1,MAPK14,MAPK3,MAPK8,MCL1,MDM2,MET,MGAM,MMP2,MMP9,MPO,MTTP,MYC,NCF1,NCOA1,NCOA2,NFE2L2,NFKBIA,NOS2,NOS3,NQO1,NR1I2,NR3C2,NUF2,ODC1,PARP1,PCNA,PGR,PIM1,PLAT,PLAU,PON1,POR,PPARA,PPARD,PPARG,PRKCA,PRKCB,PSMD3,PTGER3,PTGS2,RB1,RELA,RUNX1T1,RUNX2,RXRA,SCN5A,SELE,SERPINE1,SLC2A4,SLC6A2,SLC6A3,SLC6A4,SOAT1,SOD1,SPP1,SREBF1,STAT1,TGFB1,THBD,TNF,TOP1,TOP2A,TP53,TYR,UGT1A8,VCAM1,VEGFA,XDH,XIAP |
| GO:0005634 | nucleus | 94 | 6892 | 1.41E-05 | Cellular component | ABCG2,ACACA,ACHE,ADRB2,AHR,AKT1,ALOX5,AR,BAX,BCL2,BCL2L1,BEX2,BIRC5,CASP3,CASP7,CASP8,CASP9,CCNA2,CCNB1,CCND1,CDK1,CDK2,CDK4,CDKN1A,CHEK1,CHEK2,CHUK,DRD1,E2F1,E2F2,EGFR,ELK1,ERBB2,ESR1,ESR2,FOS,GJA1,GSK3B,GSTP1,HIF1A,HMOX1,HSF1,HSP90AA1,HSPA5,HSPB1,IGFBP3,IKBKB,IRF1,JUN,KDR,MAP2,MAPK1,MAPK14,MAPK3,MAPK8,MCL1,MDM2,MMP2,MPO,MYC,NCOA1,NCOA2,NFE2L2,NFKBIA,NOS2,NOS3,NR1I2,NR3C2,NUF2,PARP1,PCNA,PGR,PIM1,PPARA,PPARD,PPARG,PRKCA,PRKCB,PSMD3,PTGER3,RB1,RELA,RUNX1T1,RUNX2,RXRA,SOD1,SREBF1,STAT1,TGFB1,TOP1,TOP2A,TP53,TYR,XIAP |
| GO:0016020 | membrane | 104 | 8420 | 0.00022 | Cellular component | ABCC1,ABCG2,ACHE,ADCY2,ADRA2A,ADRB2,AKT1,ALOX5,APOB,AR,BAD,BAX,BCL2,BCL2L1,CA2,CASP3,CASP8,CAT,CAV1,CCNB1,CCND1,CD40LG,CDK1,CDK4,CHRNA2,CHUK,CLDN4,CTSD,CXCL10,CYP19A1,CYP1A1,CYP1A2,CYP1B1,CYP3A4,DGAT2,DPP4,DRD1,DRD2,DRD3,DUOX2,EGF,EGFR,ERBB2,ERBB3,ESR1,F3,FASN,FOS,GJA1,GSK3B,GSR,GSTP1,HK2,HMGCR,HMOX1,HSP90AA1,HSPA5,HSPB1,ICAM1,IKBKB,IL6,INSR,KCNH2,KDR,LDLR,MAOA,MAOB,MAPK1,MAPK3,MCL1,MDM2,MET,MGAM,MMP2,NCF1,NCOA1,NFE2L2,NFKBIA,NOS3,NR3C2,PGR,PIM1,PLAU,POR,PRKCA,PRKCB,PTGER3,PTGS2,SCN5A,SELE,SERPINE1,SLC2A4,SLC6A2,SLC6A3,SLC6A4,SOAT1,SREBF1,TGFB1,THBD,TNF,TYR,UGT1A8,VCAM1,VEGFA |
| GO:0042221 | response to chemical | 152 | 4153 | 1.58E-70 | Biological process | ABAT,ABCC1,ABCG2,ACACA,ADCY2,ADIPOQ,ADRA2A,ADRB2,AHR,AKT1,ALOX5,APOB,AR,BAD,BAX,BCL2,BCL2L1,BIRC5,CA2,CASP3,CASP7,CASP8,CASP9,CAT,CAV1,CCL2,CCNA2,CCNB1,CCND1,CD40LG,CDK1,CDK2,CDK4,CDKN1A,CHEK2,CHRNA2,CHUK,CLDN4,COL1A1,COL3A1,CXCL10,CXCL2,CXCL8,CYP1A1,CYP1A2,CYP1B1,CYP3A4,DGAT2,DRD1,DRD2,DRD3,DUOX2,E2F1,EGFR,ELK1,ERBB2,ESR1,ESR2,F3,FASN,FOS,GJA1,GSK3B,GSR,GSTM1,GSTP1,HIF1A,HK2,HMGCR,HMOX1,HSF1,HSP90AA1,HSPA5,HSPB1,ICAM1,IFNG,IGF2,IKBKB,IL10,IL1A,IL1B,IL2,IL4,IL6,INSR,IRF1,JUN,KCNH2,KDR,LDLR,MAOA,MAOB,MAP2,MAPK1,MAPK14,MAPK3,MAPK8,MCL1,MDM2,MET,MMP1,MMP2,MMP3,MMP9,MPO,MYC,NCF1,NCOA1,NCOA2,NFE2L2,NFKBIA,NOS2,NOS3,NQO1,NR1I2,NR3C2,PARP1,PCNA,PGR,PIM1,PLAU,PON1,POR,PPARA,PPARD,PPARG,PRKCA,PTGS2,RB1,RELA,RUNX2,RXRA,SCN5A,SELE,SERPINE1,SLC2A4,SLC6A2,SLC6A3,SLC6A4,SOD1,SPP1,SREBF1,STAT1,TGFB1,THBD,TNF,TOP1,TP53,TYR,UGT1A8,VCAM1,VEGFA |
| GO:0050896 | response to stimulus | 169 | 7824 | 3.36E-52 | Biological process | ABAT,ABCC1,ABCG2,ACACA,ACHE,ADCY2,ADIPOQ,ADRA2A,ADRB2,AHR,AHSA1,AKT1,ALOX5,APOB,AR,BAD,BAX,BCL2,BCL2L1,BIRC5,CA2,CASP3,CASP7,CASP8,CASP9,CAT,CAV1,CCL2,CCNA2,CCNB1,CCND1,CD40LG,CDK1,CDK2,CDK4,CDKN1A,CHEK1,CHEK2,CHRNA2,CHUK,CLDN4,COL1A1,COL3A1,CRP,CTSD,CXCL10,CXCL2,CXCL8,CYP1A1,CYP1A2,CYP1B1,CYP3A4,DGAT2,DPP4,DRD1,DRD2,DRD3,DUOX2,E2F1,E2F2,EGF,EGFR,ELK1,ERBB2,ERBB3,ESR1,ESR2,F3,FASN,FOS,GJA1,GSK3B,GSR,GSTM1,GSTP1,HIF1A,HK2,HMGCR,HMOX1,HSF1,HSP90AA1,HSPA5,HSPB1,ICAM1,IFNG,IGF2,IKBKB,IL10,IL1A,IL1B,IL2,IL4,IL6,INSR,IRF1,JUN,KCNH2,KDR,LDLR,MAOA,MAOB,MAP2,MAPK1,MAPK14,MAPK3,MAPK8,MCL1,MDM2,MET,MGAM,MMP1,MMP2,MMP3,MMP9,MPO,MYC,NCF1,NCOA1,NCOA2,NFE2L2,NFKBIA,NOS2,NOS3,NQO1,NR1I2,NR3C2,ODC1,PARP1,PCNA,PGR,PIM1,PLAT,PLAU,PON1,POR,PPARA,PPARD,PPARG,PRKCA,PRKCB,PSMD3,PTGER3,PTGS2,RB1,RELA,RUNX2,RXRA,SCN5A,SELE,SERPINE1,SLC2A4,SLC6A2,SLC6A3,SLC6A4,SOD1,SPP1,SREBF1,STAT1,TGFB1,THBD,TNF,TOP1,TOP2A,TP53,TYR,UGT1A8,VCAM1,VEGFA,XIAP |
| GO:0048518 | positive regulation of biological process | 148 | 5459 | 7.50E-50 | Biological process | ABAT,ACACA,ACHE,ADIPOQ,ADRA2A,ADRB2,AHR,AKT1,APOB,AR,BAD,BAX,BCL2,BCL2L1,BIRC5,CA2,CASP3,CASP8,CASP9,CAT,CAV1,CCL2,CCNA2,CCNB1,CCND1,CD40LG,CDK1,CDK2,CDK4,CDKN1A,CHEK1,CHEK2,CHUK,COL1A1,COL3A1,CRP,CXCL10,CXCL2,CXCL8,CYP19A1,CYP1A1,CYP1B1,DGAT2,DPP4,DRD1,DRD2,DRD3,E2F1,E2F2,EGF,EGFR,ELK1,ERBB2,ERBB3,ESR1,ESR2,F3,FASN,FOS,GJA1,GSK3B,GSTP1,HIF1A,HK2,HMGCR,HMOX1,HSF1,HSP90AA1,HSPA5,HSPB1,ICAM1,IFNG,IGF2,IGFBP3,IKBKB,IL10,IL1A,IL1B,IL2,IL4,IL6,INSR,IRF1,JUN,KCNH2,KDR,LDLR,MAOB,MAP2,MAPK1,MAPK14,MAPK3,MAPK8,MCL1,MDM2,MET,MMP1,MMP2,MMP3,MMP9,MYC,NCF1,NCOA1,NCOA2,NFE2L2,NFKBIA,NOS2,NOS3,NQO1,NR1I2,ODC1,PARP1,PCNA,PGR,PIM1,PLAU,PON1,POR,PPARA,PPARD,PPARG,PRKCA,PRKCB,PTGER3,PTGS2,RB1,RELA,RUNX2,RXRA,SCN5A,SELE,SERPINE1,SLC6A3,SLC6A4,SOAT1,SOD1,SPP1,SREBF1,STAT1,TGFB1,THBD,TNF,TOP2A,TP53,VCAM1,VEGFA,XDH,XIAP |
| GO:0051716 | cellular response to stimulus | 152 | 6212 | 1.21E-46 | Biological process | ABCC1,ACACA,ADCY2,ADIPOQ,ADRA2A,ADRB2,AHR,AKT1,ALOX5,APOB,AR,BAD,BAX,BCL2,BCL2L1,BIRC5,CA2,CASP3,CASP7,CASP8,CASP9,CAT,CAV1,CCL2,CCNA2,CCNB1,CCND1,CD40LG,CDK1,CDK2,CDK4,CDKN1A,CHEK1,CHEK2,CHRNA2,CHUK,CLDN4,COL1A1,COL3A1,CXCL10,CXCL2,CXCL8,CYP1A1,CYP1A2,CYP1B1,CYP3A4,DGAT2,DRD1,DRD2,DRD3,DUOX2,E2F1,E2F2,EGF,EGFR,ELK1,ERBB2,ERBB3,ESR1,ESR2,F3,FASN,FOS,GJA1,GSK3B,GSR,GSTM1,GSTP1,HIF1A,HK2,HMOX1,HSF1,HSP90AA1,HSPA5,HSPB1,ICAM1,IFNG,IGF2,IKBKB,IL10,IL1A,IL1B,IL2,IL4,IL6,INSR,IRF1,JUN,KCNH2,KDR,LDLR,MAOA,MAP2,MAPK1,MAPK14,MAPK3,MAPK8,MCL1,MDM2,MET,MMP1,MMP2,MMP3,MMP9,MPO,MYC,NCF1,NCOA1,NCOA2,NFE2L2,NFKBIA,NOS2,NOS3,NQO1,NR1I2,NR3C2,PARP1,PCNA,PGR,PIM1,PLAT,PLAU,POR,PPARA,PPARD,PPARG,PRKCA,PRKCB,PTGER3,PTGS2,RB1,RELA,RUNX2,RXRA,SCN5A,SELE,SERPINE1,SLC2A4,SLC6A4,SOD1,SPP1,SREBF1,STAT1,TGFB1,THBD,TNF,TOP2A,TP53,UGT1A8,VCAM1,VEGFA,XIAP |
| GO:0065007 | biological regulation | 171 | 11740 | 1.80E-27 | Biological process | ABAT,ABCC1,ABCG2,ACACA,ACHE,ADCY2,ADIPOQ,ADRA2A,ADRB2,AHR,AHSA1,AKT1,ALOX5,APOB,AR,BAD,BAX,BCL2,BCL2L1,BEX2,BIRC5,CA2,CASP3,CASP8,CASP9,CAT,CAV1,CCL2,CCNA2,CCNB1,CCND1,CD40LG,CDK1,CDK2,CDK4,CDKN1A,CHEK1,CHEK2,CHRNA2,CHUK,CLDN4,COL1A1,COL3A1,CRP,CXCL10,CXCL2,CXCL8,CYP19A1,CYP1A1,CYP1A2,CYP1B1,CYP3A4,DGAT2,DPP4,DRD1,DRD2,DRD3,DUOX2,E2F1,E2F2,EGF,EGFR,ELK1,ERBB2,ERBB3,ESR1,ESR2,F3,FASN,FOS,GJA1,GSK3B,GSR,GSTP1,HIF1A,HK2,HMGCR,HMOX1,HSF1,HSP90AA1,HSPA5,HSPB1,ICAM1,IFNG,IGF2,IGFBP3,IKBKB,IL10,IL1A,IL1B,IL2,IL4,IL6,INSR,IRF1,JUN,KCNH2,KDR,LDLR,MAOA,MAOB,MAP2,MAPK1,MAPK14,MAPK3,MAPK8,MCL1,MDM2,MET,MMP1,MMP2,MMP3,MMP9,MPO,MTTP,MYC,NCF1,NCOA1,NCOA2,NFE2L2,NFKBIA,NOS2,NOS3,NQO1,NR1I2,NR3C2,ODC1,PARP1,PCNA,PGR,PIM1,PLAT,PLAU,PON1,POR,PPARA,PPARD,PPARG,PRKCA,PRKCB,PSMD3,PTGER3,PTGS2,RB1,RELA,RUNX1T1,RUNX2,RXRA,SCN5A,SELE,SERPINE1,SLC2A4,SLC6A2,SLC6A3,SLC6A4,SOAT1,SOD1,SPP1,SREBF1,STAT1,TGFB1,THBD,TNF,TOP1,TOP2A,TP53,UGT1A8,VCAM1,VEGFA,XDH,XIAP |
| GO:0050789 | regulation of biological process | 165 | 11116 | 7.77E-25 | Biological process | ABAT,ACACA,ACHE,ADCY2,ADIPOQ,ADRA2A,ADRB2,AHR,AKT1,ALOX5,APOB,AR,BAD,BAX,BCL2,BCL2L1,BEX2,BIRC5,CA2,CASP3,CASP8,CASP9,CAT,CAV1,CCL2,CCNA2,CCNB1,CCND1,CD40LG,CDK1,CDK2,CDK4,CDKN1A,CHEK1,CHEK2,CHRNA2,CHUK,CLDN4,COL1A1,COL3A1,CRP,CXCL10,CXCL2,CXCL8,CYP19A1,CYP1A1,CYP1A2,CYP1B1,DGAT2,DPP4,DRD1,DRD2,DRD3,DUOX2,E2F1,E2F2,EGF,EGFR,ELK1,ERBB2,ERBB3,ESR1,ESR2,F3,FASN,FOS,GJA1,GSK3B,GSR,GSTP1,HIF1A,HK2,HMGCR,HMOX1,HSF1,HSP90AA1,HSPA5,HSPB1,ICAM1,IFNG,IGF2,IGFBP3,IKBKB,IL10,IL1A,IL1B,IL2,IL4,IL6,INSR,IRF1,JUN,KCNH2,KDR,LDLR,MAOA,MAOB,MAP2,MAPK1,MAPK14,MAPK3,MAPK8,MCL1,MDM2,MET,MMP1,MMP2,MMP3,MMP9,MPO,MTTP,MYC,NCF1,NCOA1,NCOA2,NFE2L2,NFKBIA,NOS2,NOS3,NQO1,NR1I2,NR3C2,ODC1,PARP1,PCNA,PGR,PIM1,PLAT,PLAU,PON1,POR,PPARA,PPARD,PPARG,PRKCA,PRKCB,PSMD3,PTGER3,PTGS2,RB1,RELA,RUNX1T1,RUNX2,RXRA,SCN5A,SELE,SERPINE1,SLC6A3,SLC6A4,SOAT1,SOD1,SPP1,SREBF1,STAT1,TGFB1,THBD,TNF,TOP1,TOP2A,TP53,UGT1A8,VCAM1,VEGFA,XDH,XIAP |
| GO:0050794 | regulation of cellular process | 160 | 10484 | 6.57E-24 | Biological process | ABAT,ACACA,ACHE,ADCY2,ADIPOQ,ADRA2A,ADRB2,AHR,AKT1,ALOX5,APOB,AR,BAD,BAX,BCL2,BCL2L1,BEX2,BIRC5,CA2,CASP3,CASP8,CASP9,CAT,CAV1,CCL2,CCNA2,CCNB1,CCND1,CD40LG,CDK1,CDK2,CDK4,CDKN1A,CHEK1,CHEK2,CHRNA2,CHUK,CLDN4,COL1A1,COL3A1,CRP,CXCL10,CXCL2,CXCL8,CYP19A1,CYP1A1,CYP1B1,DGAT2,DPP4,DRD1,DRD2,DRD3,DUOX2,E2F1,E2F2,EGF,EGFR,ELK1,ERBB2,ERBB3,ESR1,ESR2,F3,FASN,FOS,GJA1,GSK3B,GSR,GSTP1,HIF1A,HK2,HMGCR,HMOX1,HSF1,HSP90AA1,HSPA5,HSPB1,ICAM1,IFNG,IGF2,IGFBP3,IKBKB,IL10,IL1A,IL1B,IL2,IL4,IL6,INSR,IRF1,JUN,KCNH2,KDR,LDLR,MAOA,MAOB,MAP2,MAPK1,MAPK14,MAPK3,MAPK8,MCL1,MDM2,MET,MMP1,MMP2,MMP3,MMP9,MPO,MYC,NCF1,NCOA1,NCOA2,NFE2L2,NFKBIA,NOS2,NOS3,NQO1,NR1I2,NR3C2,ODC1,PARP1,PCNA,PGR,PIM1,PLAT,PLAU,POR,PPARA,PPARD,PPARG,PRKCA,PRKCB,PTGER3,PTGS2,RB1,RELA,RUNX1T1,RUNX2,RXRA,SCN5A,SELE,SERPINE1,SLC6A3,SLC6A4,SOAT1,SOD1,SPP1,SREBF1,STAT1,TGFB1,THBD,TNF,TOP2A,TP53,UGT1A8,VCAM1,VEGFA,XDH,XIAP |
| GO:0008152 | metabolic process | 153 | 9569 | 2.22E-23 | Biological process | ABAT,ABCC1,ABCG2,ACACA,ACHE,ADCY2,ADIPOQ,ADRB2,AHR,AKT1,ALOX5,APOB,AR,BAD,BAX,BCL2,BIRC5,CASP3,CASP7,CASP8,CASP9,CAT,CAV1,CCL2,CCNA2,CCNB1,CCND1,CD40LG,CDK1,CDK2,CDK4,CDKN1A,CHEK1,CHEK2,CHUK,COL1A1,COL3A1,CTRB1,CTSD,CXCL8,CYP19A1,CYP1A1,CYP1A2,CYP1B1,CYP3A4,DGAT2,DPP4,DRD1,DRD2,DRD3,DUOX2,E2F1,E2F2,EGF,EGFR,ELK1,ERBB2,ERBB3,ESR1,ESR2,F3,FASN,FOS,GSK3B,GSR,GSTM1,GSTP1,HIF1A,HK2,HMGCR,HMOX1,HSF1,HSP90AA1,HSPA5,IGF2,IGFBP3,IKBKB,IL10,IL1B,IL2,IL4,IL6,INSR,IRF1,JUN,KCNH2,KDR,LDLR,MAOA,MAOB,MAPK1,MAPK14,MAPK3,MAPK8,MDM2,MET,MGAM,MMP1,MMP2,MMP3,MMP9,MPO,MTTP,MYC,NCF1,NCOA1,NCOA2,NFE2L2,NFKBIA,NOS2,NOS3,NQO1,NR1I2,NR3C2,ODC1,PARP1,PCNA,PGR,PIM1,PLAT,PLAU,PON1,POR,PPARA,PPARD,PPARG,PRKCA,PRKCB,PSMD3,PTGS2,RB1,RELA,RUNX1T1,RUNX2,RXRA,SLC2A4,SLC6A3,SLC6A4,SOAT1,SOD1,SPP1,SREBF1,STAT1,TGFB1,TNF,TOP1,TOP2A,TP53,TYR,UGT1A8,VCAM1,XDH,XIAP |
| GO:0071704 | organic substance metabolic process | 149 | 9135 | 7.94E-23 | Biological process | ABAT,ABCC1,ABCG2,ACACA,ACHE,ADCY2,ADIPOQ,ADRB2,AHR,AKT1,ALOX5,APOB,AR,BAD,BAX,BCL2,BIRC5,CASP3,CASP7,CASP8,CASP9,CAT,CAV1,CCL2,CCNA2,CCNB1,CCND1,CD40LG,CDK1,CDK2,CDK4,CDKN1A,CHEK1,CHEK2,CHUK,COL3A1,CTRB1,CTSD,CXCL8,CYP19A1,CYP1A1,CYP1A2,CYP1B1,CYP3A4,DGAT2,DPP4,DRD1,DRD2,DRD3,DUOX2,E2F1,E2F2,EGF,EGFR,ELK1,ERBB2,ERBB3,ESR1,ESR2,F3,FASN,FOS,GSK3B,GSR,GSTM1,GSTP1,HIF1A,HK2,HMGCR,HMOX1,HSF1,HSP90AA1,HSPA5,IGF2,IGFBP3,IKBKB,IL10,IL1B,IL2,IL4,IL6,INSR,IRF1,JUN,KCNH2,KDR,LDLR,MAOA,MAOB,MAPK1,MAPK14,MAPK3,MAPK8,MDM2,MET,MGAM,MMP1,MMP2,MMP3,MMP9,MPO,MTTP,MYC,NCOA1,NCOA2,NFE2L2,NFKBIA,NOS2,NOS3,NR1I2,NR3C2,ODC1,PARP1,PCNA,PGR,PIM1,PLAT,PLAU,PON1,POR,PPARA,PPARD,PPARG,PRKCA,PRKCB,PSMD3,PTGS2,RB1,RELA,RUNX1T1,RUNX2,RXRA,SLC2A4,SLC6A3,SOAT1,SOD1,SPP1,SREBF1,STAT1,TGFB1,TNF,TOP1,TOP2A,TP53,TYR,UGT1A8,VCAM1,XDH,XIAP |
| GO:0009987 | cellular process | 176 | 14652 | 2.79E-18 | Biological process | ABAT,ABCC1,ABCG2,ACACA,ACHE,ADCY2,ADIPOQ,ADRA2A,ADRB2,AHR,AKT1,ALOX5,APOB,AR,BAD,BAX,BCL2,BCL2L1,BEX2,BIRC5,CA2,CASP3,CASP7,CASP8,CASP9,CAT,CAV1,CCL2,CCNA2,CCNB1,CCND1,CD40LG,CDK1,CDK2,CDK4,CDKN1A,CHEK1,CHEK2,CHRNA2,CHUK,CLDN4,COL1A1,COL3A1,CTRB1,CTSD,CXCL10,CXCL2,CXCL8,CYP19A1,CYP1A1,CYP1A2,CYP1B1,CYP3A4,DGAT2,DPP4,DRD1,DRD2,DRD3,DUOX2,E2F1,E2F2,EGF,EGFR,ELK1,ERBB2,ERBB3,ESR1,ESR2,F3,FASN,FOS,GJA1,GSK3B,GSR,GSTM1,GSTP1,HIF1A,HK2,HMGCR,HMOX1,HSF1,HSP90AA1,HSPA5,HSPB1,ICAM1,IFNG,IGF2,IGFBP3,IKBKB,IL10,IL1A,IL1B,IL2,IL4,IL6,INSR,IRF1,JUN,KCNH2,KDR,LDLR,MAOA,MAOB,MAP2,MAPK1,MAPK14,MAPK3,MAPK8,MCL1,MDM2,MET,MGAM,MMP1,MMP2,MMP3,MMP9,MPO,MTTP,MYC,NCF1,NCOA1,NCOA2,NFE2L2,NFKBIA,NOS2,NOS3,NQO1,NR1I2,NR3C2,NUF2,ODC1,PARP1,PCNA,PGR,PIM1,PLAT,PLAU,PON1,POR,PPARA,PPARD,PPARG,PRKCA,PRKCB,PSMD3,PTGER3,PTGS2,RB1,RELA,RUNX1T1,RUNX2,RXRA,SCN5A,SELE,SERPINE1,SLC2A4,SLC6A2,SLC6A3,SLC6A4,SOAT1,SOD1,SPP1,SREBF1,STAT1,TGFB1,THBD,TNF,TOP1,TOP2A,TP53,TYR,UGT1A8,VCAM1,VEGFA,XDH,XIAP |
| GO:0005515 | protein binding | 147 | 6607 | 1.29E-37 | Molecular function | ABAT,ABCG2,ACACA,ACHE,ADCY2,ADIPOQ,ADRA2A,ADRB2,AHR,AHSA1,AKT1,APOB,AR,BAD,BAX,BCL2,BCL2L1,BIRC5,CASP3,CASP8,CASP9,CAT,CAV1,CCL2,CCNA2,CCNB1,CCND1,CD40LG,CDK1,CDK2,CDK4,CDKN1A,CHEK1,CHEK2,CHUK,CLDN4,COL1A1,COL3A1,CRP,CXCL10,CXCL2,CXCL8,CYP1A1,CYP1A2,CYP3A4,DGAT2,DPP4,DRD2,DRD3,E2F1,E2F2,EGF,EGFR,ELK1,ERBB2,ERBB3,ESR1,ESR2,F3,FASN,FOS,GJA1,GSK3B,GSTM1,GSTP1,HIF1A,HMGCR,HMOX1,HSF1,HSP90AA1,HSPA5,HSPB1,ICAM1,IFNG,IGF2,IGFBP3,IKBKB,IL10,IL1A,IL1B,IL2,IL4,IL6,INSR,JUN,KCNH2,KDR,LDLR,MAOB,MAP2,MAPK1,MAPK14,MAPK3,MAPK8,MCL1,MDM2,MET,MMP9,MTTP,MYC,NCF1,NCOA1,NCOA2,NFE2L2,NFKBIA,NOS2,NOS3,NQO1,ODC1,PARP1,PCNA,PGR,PIM1,PLAT,PON1,POR,PPARA,PPARD,PPARG,PRKCA,PRKCB,PTGS2,RB1,RELA,RUNX2,RXRA,SCN5A,SELE,SERPINE1,SLC6A2,SLC6A3,SLC6A4,SOD1,SPP1,SREBF1,STAT1,TGFB1,TNF,TOP1,TOP2A,TP53,TYR,UGT1A8,VCAM1,VEGFA,XDH,XIAP |
| GO:0019899 | enzyme binding | 84 | 2197 | 5.32E-30 | Molecular function | ABAT,ADCY2,ADRA2A,ADRB2,AKT1,APOB,AR,BAD,BCL2,BCL2L1,BIRC5,CASP3,CASP8,CASP9,CAT,CAV1,CCNA2,CCNB1,CCND1,CDKN1A,CHEK2,COL1A1,COL3A1,CYP1A1,CYP1A2,CYP3A4,DPP4,E2F1,EGF,EGFR,ERBB2,ERBB3,ESR1,ESR2,F3,GJA1,GSK3B,GSTM1,GSTP1,HIF1A,HMGCR,HMOX1,HSF1,HSP90AA1,HSPA5,HSPB1,IKBKB,IL2,JUN,KCNH2,LDLR,MAPK1,MAPK14,MAPK3,MAPK8,MDM2,MET,NCOA1,NFKBIA,PARP1,PCNA,PGR,POR,PPARA,PPARG,PRKCA,PRKCB,PTGS2,RB1,RELA,RXRA,SCN5A,SELE,SERPINE1,SLC6A3,SLC6A4,SOD1,SREBF1,STAT1,TGFB1,TNF,TOP2A,TP53,UGT1A8 |
| GO:0042802 | identical protein binding | 74 | 1754 | 2.85E-28 | Molecular function | ABAT,ABCG2,ACACA,ACHE,ADIPOQ,ADRA2A,ADRB2,AHR,AKT1,BAX,BCL2,BCL2L1,BIRC5,CASP8,CASP9,CAT,CAV1,CHEK2,CHUK,CLDN4,COL1A1,CRP,DGAT2,DPP4,DRD2,EGFR,ERBB2,ERBB3,ESR1,ESR2,FASN,GSTM1,HMGCR,HMOX1,HSF1,HSP90AA1,HSPB1,IKBKB,INSR,JUN,KCNH2,KDR,LDLR,MAOB,MAPK1,MAPK3,MCL1,MDM2,MET,MMP9,NFKBIA,NOS2,NQO1,ODC1,PARP1,PCNA,PGR,PON1,PPARG,PTGS2,RB1,RELA,SLC6A4,SOD1,STAT1,TGFB1,TNF,TOP2A,TP53,TYR,UGT1A8,VEGFA,XDH,XIAP |
| GO:0046983 | protein dimerization activity | 64 | 1301 | 2.41E-27 | Molecular function | ABAT,ABCG2,ACHE,ADCY2,ADIPOQ,ADRA2A,ADRB2,AHR,AKT1,AR,BAD,BAX,BCL2,BCL2L1,BIRC5,CAT,CAV1,CHEK2,CHUK,DGAT2,DPP4,DRD2,E2F1,E2F2,EGFR,ERBB2,ERBB3,FASN,FOS,GSTM1,HIF1A,HMGCR,HMOX1,HSF1,HSP90AA1,HSPB1,IKBKB,JUN,KCNH2,MAOB,MCL1,MTTP,MYC,NCOA1,NCOA2,NOS2,ODC1,PON1,PPARD,PPARG,PTGS2,RELA,RXRA,SLC6A4,SOD1,SREBF1,STAT1,TGFB1,TOP2A,TP53,TYR,UGT1A8,VEGFA,XDH |
| GO:0005488 | binding | 168 | 11878 | 5.92E-23 | Molecular function | ABAT,ABCC1,ABCG2,ACACA,ACHE,ADCY2,ADIPOQ,ADRA2A,ADRB2,AHR,AHSA1,AKT1,ALOX5,APOB,AR,BAD,BAX,BCL2,BCL2L1,BIRC5,CA2,CASP3,CASP8,CASP9,CAT,CAV1,CCL2,CCNA2,CCNB1,CCND1,CD40LG,CDK1,CDK2,CDK4,CDKN1A,CHEK1,CHEK2,CHRNA2,CHUK,CLDN4,COL1A1,COL3A1,CRP,CXCL10,CXCL2,CXCL8,CYP19A1,CYP1A1,CYP1A2,CYP1B1,CYP3A4,DGAT2,DPP4,DRD1,DRD2,DRD3,DUOX2,E2F1,E2F2,EGF,EGFR,ELK1,ERBB2,ERBB3,ESR1,ESR2,F3,FASN,FOS,GJA1,GSK3B,GSR,GSTM1,GSTP1,HIF1A,HK2,HMGCR,HMOX1,HSF1,HSP90AA1,HSPA5,HSPB1,ICAM1,IFNG,IGF2,IGFBP3,IKBKB,IL10,IL1A,IL1B,IL2,IL4,IL6,INSR,IRF1,JUN,KCNH2,KDR,LDLR,MAOB,MAP2,MAPK1,MAPK14,MAPK3,MAPK8,MCL1,MDM2,MET,MGAM,MMP1,MMP2,MMP3,MMP9,MPO,MTTP,MYC,NCF1,NCOA1,NCOA2,NFE2L2,NFKBIA,NOS2,NOS3,NQO1,NR1I2,NR3C2,ODC1,PARP1,PCNA,PGR,PIM1,PLAT,PON1,POR,PPARA,PPARD,PPARG,PRKCA,PRKCB,PTGS2,RB1,RELA,RUNX1T1,RUNX2,RXRA,SCN5A,SELE,SERPINE1,SLC6A2,SLC6A3,SLC6A4,SOAT1,SOD1,SPP1,SREBF1,STAT1,TGFB1,THBD,TNF,TOP1,TOP2A,TP53,TYR,UGT1A8,VCAM1,VEGFA,XDH,XIAP |
| GO:0043167 | ion binding | 106 | 6066 | 3.52E-13 | Molecular function | ABAT,ABCC1,ABCG2,ACACA,ACHE,ADCY2,ADIPOQ,AKT1,ALOX5,APOB,AR,BAD,BIRC5,CA2,CAT,CDK1,CDK2,CDK4,CDKN1A,CHEK1,CHEK2,CHRNA2,CHUK,COL1A1,COL3A1,CRP,CXCL10,CYP19A1,CYP1A1,CYP1A2,CYP1B1,CYP3A4,DRD1,DRD2,DRD3,DUOX2,EGF,EGFR,ERBB2,ERBB3,ESR1,ESR2,F3,FASN,GSK3B,GSR,GSTM1,GSTP1,HK2,HMGCR,HMOX1,HSP90AA1,HSPA5,IGFBP3,IKBKB,IL1A,INSR,KDR,LDLR,MAOB,MAPK1,MAPK14,MAPK3,MAPK8,MDM2,MET,MMP1,MMP2,MMP3,MMP9,MPO,NCF1,NOS2,NOS3,NR1I2,NR3C2,PARP1,PGR,PIM1,PON1,POR,PPARA,PPARD,PPARG,PRKCA,PRKCB,PTGS2,RELA,RUNX1T1,RUNX2,RXRA,SELE,SLC6A2,SLC6A3,SLC6A4,SOAT1,SOD1,THBD,TOP1,TOP2A,TP53,TYR,UGT1A8,VEGFA,XDH,XIAP |
| GO:0097159 | organic cyclic compound binding | 95 | 5382 | 1.99E-11 | Molecular function | ABAT,ABCC1,ABCG2,ACACA,ADCY2,ADRA2A,ADRB2,AHR,AKT1,AR,BCL2,CAT,CAV1,CDK1,CDK2,CDK4,CHEK1,CHEK2,CHUK,CYP19A1,CYP1A1,CYP1A2,CYP1B1,CYP3A4,DRD1,DRD2,DRD3,DUOX2,E2F1,E2F2,EGFR,ELK1,ERBB2,ERBB3,ESR1,ESR2,FASN,FOS,GSK3B,GSR,HIF1A,HK2,HMGCR,HMOX1,HSF1,HSP90AA1,HSPA5,IKBKB,INSR,IRF1,JUN,KDR,MAOB,MAPK1,MAPK14,MAPK3,MAPK8,MDM2,MET,MPO,MYC,NCOA1,NCOA2,NFE2L2,NOS2,NOS3,NR1I2,NR3C2,PARP1,PCNA,PGR,PIM1,POR,PPARA,PPARD,PPARG,PRKCA,PRKCB,PTGS2,RB1,RELA,RUNX1T1,RUNX2,RXRA,SLC6A3,SLC6A4,SOAT1,SREBF1,STAT1,TNF,TOP1,TOP2A,TP53,UGT1A8,XDH |
| GO:0003824 | catalytic activity | 93 | 5592 | 1.04E-09 | Molecular function | ABAT,ABCC1,ABCG2,ACACA,ACHE,ADCY2,AKT1,ALOX5,BIRC5,CA2,CASP3,CASP7,CASP8,CASP9,CAT,CCL2,CCNB1,CCND1,CDK1,CDK2,CDK4,CDKN1A,CHEK1,CHEK2,CHUK,CTRB1,CTSD,CYP19A1,CYP1A1,CYP1A2,CYP1B1,CYP3A4,DGAT2,DPP4,DUOX2,EGF,EGFR,ERBB2,ERBB3,ESR1,FASN,GSK3B,GSR,GSTM1,GSTP1,HK2,HMGCR,HMOX1,HSP90AA1,HSPA5,IKBKB,INSR,KCNH2,KDR,MAOA,MAOB,MAPK1,MAPK14,MAPK3,MAPK8,MDM2,MET,MGAM,MMP1,MMP2,MMP3,MMP9,MPO,NCF1,NCOA1,NOS2,NOS3,NQO1,ODC1,PARP1,PCNA,PIM1,PLAT,PLAU,PON1,POR,PRKCA,PRKCB,PTGS2,SOAT1,SOD1,TOP1,TOP2A,TYR,UGT1A8,VCAM1,XDH,XIAP |
| GO:1901363 | heterocyclic compound binding | 87 | 5305 | 1.26E-08 | Molecular function | ABAT,ABCC1,ABCG2,ACACA,ADCY2,AHR,AKT1,AR,BCL2,CAT,CDK1,CDK2,CDK4,CHEK1,CHEK2,CHUK,CYP19A1,CYP1A1,CYP1A2,CYP1B1,CYP3A4,DUOX2,E2F1,E2F2,EGFR,ELK1,ERBB2,ERBB3,ESR1,ESR2,FASN,FOS,GSK3B,GSR,HIF1A,HK2,HMGCR,HMOX1,HSF1,HSP90AA1,HSPA5,IKBKB,INSR,IRF1,JUN,KDR,MAOB,MAPK1,MAPK14,MAPK3,MAPK8,MDM2,MET,MPO,MYC,NCOA1,NCOA2,NFE2L2,NOS2,NOS3,NR1I2,NR3C2,PARP1,PCNA,PGR,PIM1,POR,PPARA,PPARD,PPARG,PRKCA,PRKCB,PTGS2,RB1,RELA,RUNX1T1,RUNX2,RXRA,SLC6A4,SOAT1,SREBF1,STAT1,TNF,TOP1,TOP2A,TP53,XDH |
| GO:0043169 | cation binding | 66 | 4170 | 1.24E-05 | Molecular function | ABAT,ACACA,ACHE,ADCY2,ALOX5,AR,BIRC5,CA2,CAT,CDK2,CDKN1A,CHEK2,CHRNA2,COL1A1,COL3A1,CRP,CYP19A1,CYP1A1,CYP1A2,CYP1B1,CYP3A4,DRD1,DRD2,DRD3,DUOX2,EGF,ESR1,ESR2,HMOX1,HSPA5,IGFBP3,IL1A,LDLR,MDM2,MMP1,MMP2,MMP3,MMP9,MPO,NOS2,NOS3,NR1I2,NR3C2,PARP1,PGR,PIM1,PON1,PPARA,PPARD,PPARG,PRKCA,PRKCB,PTGS2,RUNX1T1,RXRA,SELE,SLC6A2,SLC6A3,SLC6A4,SOD1,THBD,TOP2A,TP53,TYR,XDH,XIAP |

**Table S4.** KEGG enrichment analysis.

| term ID | term description | observed gene count | background gene count | false discovery rate | matching proteins in your network (labels) |
| --- | --- | --- | --- | --- | --- |
| hsa04657 | IL-17 signaling pathway | 27 | 92 | 6.78E-29 | CASP3,CASP8,CCL2,CHUK,CXCL10,CXCL2,CXCL8,FOS,GSK3B,HSP90AA1,IFNG,IKBKB,IL1B,IL4,IL6,JUN,MAPK1,MAPK14,MAPK3,MAPK8,MMP1,MMP3,MMP9,NFKBIA,PTGS2,RELA,TNF |
| hsa04668 | TNF signaling pathway | 26 | 108 | 4.70E-26 | AKT1,CASP3,CASP7,CASP8,CCL2,CHUK,CXCL10,CXCL2,FOS,ICAM1,IKBKB,IL1B,IL6,JUN,MAPK1,MAPK14,MAPK3,MAPK8,MMP3,MMP9,NFKBIA,PTGS2,RELA,SELE,TNF,VCAM1 |
| hsa04151 | PI3K-Akt signaling pathway | 37 | 348 | 4.83E-26 | AKT1,BAD,BCL2,BCL2L1,CASP9,CCND1,CDK2,CDK4,CDKN1A,CHUK,COL1A1,EGF,EGFR,ERBB2,ERBB3,GSK3B,HSP90AA1,IGF2,IKBKB,IL2,IL4,IL6,INSR,KDR,MAPK1,MAPK3,MCL1,MDM2,MET,MYC,NOS3,PRKCA,RELA,RXRA,SPP1,TP53,VEGFA |
| hsa04218 | Cellular senescence | 26 | 156 | 8.68E-23 | AKT1,CCNA2,CCNB1,CCND1,CDK1,CDK2,CDK4,CDKN1A,CHEK1,CHEK2,CXCL8,E2F1,E2F2,IGFBP3,IL1A,IL6,MAPK1,MAPK14,MAPK3,MDM2,MYC,RB1,RELA,SERPINE1,TGFB1,TP53 |
| hsa04210 | Apoptosis | 24 | 135 | 1.27E-21 | AKT1,BAD,BAX,BCL2,BCL2L1,CASP3,CASP7,CASP8,CASP9,CHUK,CTSD,FOS,IKBKB,JUN,MAPK1,MAPK3,MAPK8,MCL1,NFKBIA,PARP1,RELA,TNF,TP53,XIAP |
| hsa04010 | MAPK signaling pathway | 30 | 293 | 7.42E-21 | AKT1,CASP3,CHUK,EGF,EGFR,ELK1,ERBB2,ERBB3,FOS,HSPB1,IGF2,IKBKB,IL1A,IL1B,INSR,JUN,KDR,MAPK1,MAPK14,MAPK3,MAPK8,MET,MYC,PRKCA,PRKCB,RELA,TGFB1,TNF,TP53,VEGFA |
| hsa04066 | HIF-1 signaling pathway | 21 | 98 | 1.72E-20 | AKT1,BCL2,CDKN1A,EGF,EGFR,ERBB2,HIF1A,HK2,HMOX1,IFNG,IL6,INSR,MAPK1,MAPK3,NOS2,NOS3,PRKCA,PRKCB,RELA,SERPINE1,VEGFA |
| hsa04659 | Th17 cell differentiation | 21 | 102 | 3.43E-20 | AHR,CHUK,FOS,HIF1A,HSP90AA1,IFNG,IKBKB,IL1B,IL2,IL4,IL6,JUN,MAPK1,MAPK14,MAPK3,MAPK8,NFKBIA,RELA,RXRA,STAT1,TGFB1 |
| hsa05164 | Influenza A | 23 | 168 | 1.32E-18 | AKT1,CASP9,CCL2,CXCL10,CXCL8,GSK3B,ICAM1,IFNG,IKBKB,IL1A,IL1B,IL6,JUN,MAPK1,MAPK14,MAPK3,MAPK8,NFKBIA,PRKCA,PRKCB,RELA,STAT1,TNF |
| hsa04926 | Relaxin signaling pathway | 21 | 130 | 2.41E-18 | ADCY2,AKT1,COL1A1,COL3A1,EGFR,FOS,JUN,MAPK1,MAPK14,MAPK3,MAPK8,MMP1,MMP2,MMP9,NFKBIA,NOS2,NOS3,PRKCA,RELA,TGFB1,VEGFA |
| hsa04620 | Toll-like receptor signaling pathway | 19 | 102 | 1.15E-17 | AKT1,CASP8,CHUK,CXCL10,CXCL8,FOS,IKBKB,IL1B,IL6,JUN,MAPK1,MAPK14,MAPK3,MAPK8,NFKBIA,RELA,SPP1,STAT1,TNF |
| hsa04068 | FoxO signaling pathway | 20 | 130 | 3.45E-17 | AKT1,CAT,CCNB1,CCND1,CDK2,CDKN1A,CHUK,EGF,EGFR,IKBKB,IL10,IL6,INSR,MAPK1,MAPK14,MAPK3,MAPK8,MDM2,SLC2A4,TGFB1 |
| hsa04621 | NOD-like receptor signaling pathway | 21 | 166 | 1.68E-16 | BCL2,BCL2L1,CASP8,CCL2,CHUK,CXCL2,CXCL8,HSP90AA1,IKBKB,IL1B,IL6,JUN,MAPK1,MAPK14,MAPK3,MAPK8,NFKBIA,RELA,STAT1,TNF,XIAP |
| hsa05203 | Viral carcinogenesis | 19 | 183 | 1.20E-13 | BAD,BAX,CASP3,CASP8,CCNA2,CCND1,CDK1,CDK2,CDK4,CDKN1A,CHEK1,JUN,MAPK1,MAPK3,MDM2,NFKBIA,RB1,RELA,TP53 |
| hsa01100 | Metabolic pathways | 22 | 1250 | 0.0039 | ABAT,ACACA,ALOX5,CYP19A1,CYP1A1,CYP1A2,CYP3A4,DGAT2,FASN,HK2,HMGCR,HMOX1,MAOA,MAOB,MGAM,NOS2,NOS3,ODC1,PTGS2,TYR,UGT1A8,XDH |
